# MicroRNA roles in signalling during lactation: an insight from differential expression, time course and pathway analyses of deep sequence data

**DOI:** 10.1038/srep44605

**Published:** 2017-03-20

**Authors:** Duy N. Do, Ran Li, Pier-Luc Dudemaine, Eveline M. Ibeagha-Awemu

**Affiliations:** 1 Agriculture and Agri-Food Canada, Sherbrooke Research and Development Centre, 2000 College Street, Sherbrooke, Quebec, J1M 0C8, Canada; 2 Department of Animal Science, McGill University, 21111, Lakeshore Road, Ste-Anne-de Bellevue, Quebec, J1M 0C8, Canada; 3 College of Animal Science and Technology, Northwest A&F University, Xinong road 22, Shaanxi, 712100, China

## Abstract

The study examined microRNA (miRNA) expression and regulatory patterns during an entire bovine lactation cycle. Total RNA from milk fat samples collected at the lactogenesis (LAC, day1 [D1] and D7), galactopoiesis (GAL, D30, D70, D130, D170 and D230) and involution (INV, D290 and when milk production dropped to 5 kg/day) stages from 9 cows was used for miRNA sequencing. A total of 475 known and 238 novel miRNAs were identified. Fifteen abundantly expressed miRNAs across lactation stages play regulatory roles in basic metabolic, cellular and immunological functions. About 344, 366 and 209 miRNAs were significantly differentially expressed (DE) between GAL and LAC, INV and GAL, and INV and LAC stages, respectively. MiR-29b/miR-363 and miR-874/miR-6254 are important mediators for transition signals from LAC to GAL and from GAL to INV, respectively. Moreover, 58 miRNAs were dynamically DE in all lactation stages and 19 miRNAs were significantly time-dependently DE throughout lactation. Relevant signalling pathways for transition between lactation stages are involved in apoptosis (PTEN and SAPK/JNK), intracellular signalling (protein kinase A, TGF-β and ERK5), cell cycle regulation (STAT3), cytokines, hormones and growth factors (prolactin, growth hormone and glucocorticoid receptor). Overall, our data suggest diverse, temporal and physiological signal-dependent regulatory and mediator functions for miRNAs during lactation.

Lactation is a dynamic process and each stage of lactation is determined by interaction of many factors such as management, nutrition, health status as well as genetics and epigenetics factors. A typical dairy lactation curve begins with a rapid increase in milk yield to peak milk production around lactation days 40–50 followed by a gradual decrease until animals are dried-off (~lactation day 305)^1^. Genetic improvement of milk production requires a comprehensive view of the biology of the lactation process, from a single stage to the entire lactation curve. Massive parallel next generation sequencing (NGS), now ascertained as a comprehensive and accurate tool for analysing complex omics systems underlying biological processes^2^, offers great opportunities to elucidate the underlying mechanisms of complex traits such as the lactation process.

MicroRNAs (miRNAs), small noncoding RNA molecules regulate gene expression post-transcriptionally and play key roles in a wide range of biological processes. The roles of miRNAs in dairy lactation process or mammary gland development have been investigated using different approaches such as microarray^3^, RNA sequencing^4^,^5^,^6^,^7^ and functional analyses of specific miRNAs^8^,^9^,^10^,^11^,^12^,^13^,^14^. For instance, Wang *et al*.^3^ used microarray to analyse miRNA expression in cows during early lactation (30 days postpartum), dry period (30 days prepartum) and fresh period (7 days postpartum) and detected twelve down-regulated miRNAs in the dry compared to the lactating period and one up-regulated miRNA (miR-31) in early lactation compared to the dry period. Another study identified 56 significantly differentially expressed miRNAs between lactation and non-lactation periods^4^. Also, roles for miRNAs in milk protein metabolism and quality have been revealed^15^. Additionally, functional validation has directly linked a number of miRNAs with regulatory roles in milk synthesis, fatty acid metabolism and cellular differentiation. Down-regulation of miR-26a/b and their host genes (*CTDSPL, CTDSP2* and *CTDSP1*) in goat mammary epithelial cells was shown to decrease the expression of genes related to fatty acid synthesis (*PPARG, LXRA, SREBF1, FASN, ACACA, GPAM, LPIN1, DGAT1* and *SCD1*), triacylglycerol accumulation and unsaturated fatty acid synthesis as well as demonstration of a functional association between miR-26a/b, their host genes and *INSIG1*^16^. MiR-155a was observed to regulate the expression of growth hormone receptor and decrease bovine mammary epithelial cell viability and lactation^12^. A role for miR-152 in the development and lactation of mammary glands in dairy cows through regulation of *DNMT1* gene has been suggested^10^. MiR-24 was shown to control triacylglycerol synthesis in goat mammary epithelial cells by targeting the *FASN* gene^17^. Overexpression of miR-30b in the developing mammary gland of transgenic mouse caused lactation defects such as reduced size of alveolar lumen, defect of the lipid droplet and growth defect in pups as well as delayed involution, thus suggesting the importance of miR-30b in mammary gland biology and how the deregulation of only one miRNA could affect lactation and involution^18^. Other miRNAs like miR-15a have been shown to inhibit the expression of caseins, epithelial cell number as well as the expression of *GHR* mRNA and protein^12^. A role for miR-103 in the control of milk fat accumulation in goat mammary gland during lactation has been demonstrated^19^. These data indicate important roles of miRNAs in mammary gland development and lactation.

However, little is known about the involvement of miRNAs in transcriptional regulation of the different biological pathways involved in the entire bovine lactation process as well as in each transition stage of lactation. In order to better understand the biology underlying the bovine lactation curve, this study examined dynamic miRNA expression patterns during lactation and at each transition stage of lactation to identify key regulatory miRNAs of the lactation process. Moreover, a time course analysis was applied to establish whether the expression of miRNAs is time dependent.

## Results

### MiRNA expression in the bovine lactation curve

Milk samples were obtained from nine cows (first to third parity) on day1 (D1) and D7 (lactogenesis [**LAC**] stage), D30, D70, D130, D170 and D230 (galactopoiesis [**GAL**] stage), D290 and when milk production dropped to 5 kg/day (D5kg) (involution [**INV**] stage)^20^,^21^,^22^ for RNA isolation from the fat layer and deep sequencing. The RIN (RNA integrity number) values of isolated RNA ranged from 2.3 to 8.5 and further evaluation showed that the small RNA portion was intact and suitable for sequencing (Figure S1). Our previous data showed that miRNA deep sequence data from milk fat layer was the closest representation of mammary gland tissue thus validating the milk fat layer as a non-invasive source of miRNA for the study of mammary gland functions^23^. The distribution of raw milk yield by day and a predicted lactation curve using Wood’s model^24^ are shown in Figure S2. Tukey’s HSD (honest significant difference) test showed that milk yield at D290 and D5kg were significantly differed from other dates (Figure S2).

Next-generation sequencing of 71 libraries from 9 cows and representing the entire bovine lactation curve generated a total of 680 million reads. After adaptor trimming and size selection, 338 million reads with length ranging from 17 to 30 nucleotides and having a phred score >20 were retained for analysis. Out of this number, 82.32% mapped to unique positions on the bovine genome (University of Maryland assembly of *B. taurus*, release 3.1 -UMD.3.1), 16.31% were unmapped while 1.37% mapped to more than 5 positions and were discarded (Table S1). Mapped reads belonging to other RNA species, tRNA (10.86%), rRNA (0.60%), snRNA (0.04%) and snoRNA (0.28%) were discarded. Length distribution of reads retained for miRNA analysis indicated that majority were around 22 nucleotides long (Table S2).

Identified known miRNAs having a total raw read count of <10 were discarded. Additionally, only known miRNAs that passed the threshold of 10 read counts per million and present in at least 7 libraries (10% of total libraries) were used in differential expression (DE) analysis (Table S3a and b). MiRdeep2 score of 5 was chosen as the cut off for novel miRNA prediction as it yielded a novel miRNA true positive rate of 89 ± 1%, false positive rate of 36 ± 2 to 58 ± 8 and an estimated signal-to noise ratio of 12.3 to 14.3 (Table S4). Further filtering (10 read counts per million and present in at least 10% of total libraries) was applied to retain only true novel miRNAs (Table S3c). Based on these criteria, a total of 475 known and 238 novel miRNAs were identified in this study (Table S3a–d). Furthermore, 13 novel miRNAs originated from two genomic locations, one from three genomic locations and one from four genomic locations (Table S3c).

**Figure 1:**
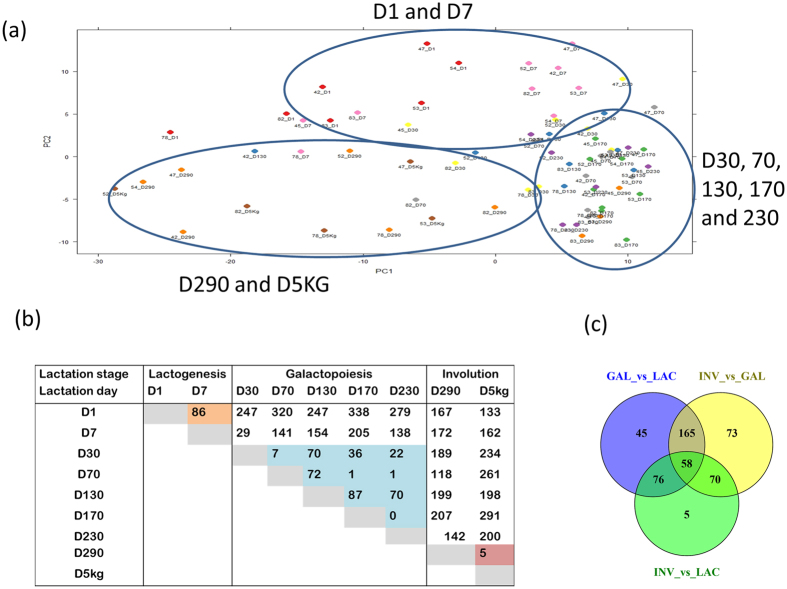
Principal component analysis (PCA) of miRNA expression showing distribution of cows based on PCA 1 and 2 (**a**), differential miRNA expression by lactation day (**b**) and differential miRNA expression by lactation stage (**c**). Differential miRNA expression between lactation days or lactation stages was declared significant at p–value BH < 0.05. LAC: Lactogenesis; GAL: Galactopoiesis and INV: Involution.

Lactation curve dynamics may be affected by miRNA expression pattern thus we carried out a principal component analysis (PCA) to understand the miRNA expression pattern of each sample as compared to all other samples. Results showed, according to component 1 and 2, a clustering of miRNA expression mainly by day (Fig. 1a). Samples of D1 and D7 clustered together, D290 and D5kg clustered together while D30 to D230 were mostly in the same cluster. Moreover, 16, 18 and 23 miRNAs were highly expressed (read counts ≥1% of total read counts) and constituted 78.2, 84.2 and 81.4% of all read counts at the LAC, GAL and INV stages, respectively (Table S5). Among them, 15 miRNAs were common to the three stages (Table 1).

MiR-148a and miR-26a were the most abundantly expressed miRNAs since they accounted for more than 10% of the read counts in each stage of lactation. The 15 commonly highly expressed miRNAs could target 5,644 unique genes with high confidence (experimentally validated or highly predicted) in Ingenuity Pathway Analysis (IPA) database. The target genes were significantly enriched in 20 molecular and cellular functions (e.g. gene expression, cell morphology, cellular assembly/organization, cellular function/maintenance and cellular development, etc.) and 22 physiological system development functions (e.g. organismal survival/development and organ development, etc.) (Table 2). The target genes were also significantly enriched in 162 canonical pathways and the five top enriched pathways were molecular mechanisms of cancer, glioblastoma multiforme, Wnt/β-catenin, protein kinase A and RhoGDI signalling (Table S6).

Moreover, a novel miRNA, miR-EIA3-33361, was the most highly expressed among novel miRNAs. MiR-EIA3-33361 could significantly target 198 unique genes enriched (significantly) for several molecular and cellular functions (e.g. cellular development/assembly/organization, molecular transport and small molecule biochemistry, etc.), physiological system development functions (e.g. tissue development and organismal development, etc.) and 15 canonical pathways (Table S7a–c). The most significantly enriched pathway was estrogen receptor signalling. Many immunological related pathways were also enriched such as tumoricidal function of hepatic natural killer cells, lymphotoxin β receptor signalling, cytotoxic T lymphocyte-mediated apoptosis of target cells, leukocyte extravasation signalling, NF-κB signalling and CD40 signalling.

### Differential miRNA expression and downstream target gene enrichment analyses

#### Differential miRNA expression between lactation days and lactation stages

The number of miRNAs significantly differentially expressed (DE) at Benjamini and Hochberg^25^ corrected p-values < 0.05 between lactation days are shown in Fig. 1b. The highest number of DE miRNAs was found between D1 and D170 (338 miRNAs), while no miRNA was DE between D170 and D230. High DE miRNAs were recorded between D1 and D30 to D5kg (range from 133 to 338 miRNAs), between D5kg and D1 to D230 (range from 133 to 291 miRNAs), between D290 and D1 to D230 (range from 118 to 207 miRNAs) and between D7 and D70 to D5kg (range from 141 to 205 miRNAs) (Fig. 1b). Consistent with PCA results, a low number of DE miRNAs was recorded between D1 and D7 (LAC stage, 86 miRNAs)), between lactation days in GAL stage (0 to 87 miRNAs) and between D290 and D5kg (INV stage, 5 miRNAs).

When comparisons were made between lactation stages, 344 (133 up- and 211 down-regulated), 366 (229 up- and 137 down-regulated) and 209 (124 up- and 85 down-regulated) miRNAs were significantly DE between GAL and LAC, INV and GAL, and INV and LAC, respectively (Fig. 1c and Table S8a–c). Among significantly DE miRNAs, 58 were common between the three comparisons and considered dynamically DE miRNAs. MiRNAs unique to each pair of comparison were 15 for GAL vs LAC, 40 for INV vs GAL and 13 for INV vs LAC. The top most significantly DE miRNAs (p-value ≤ 1.00E-12) or with a log2 fold change (|L2FC|) ≥ 3 and p-value ≤ 1.00E-5) between lactation stages are shown in Table 3 while 58 dynamically DE miRNAs are shown in Table 4. The most significantly up and down DE miRNAs between LAC and GAL were miR-29b (p-value = 2.99E-31) and miR-363 (p-value = 2.13E-22), between GAL and INV were miR-874 (p-value = 9.37E-14) and miR-6524 (p-value = 1.84E-09) and between INV and LAC were miR-885 (p-value = 1.70E-31) and miR-2285t (p-value = 7.18E-24), respectively (Tables 3 and S8a–c). The corresponding most enriched pathways were axonal guidance signalling (miR-29b), calcium signalling (miR-363), granulocyte adhesion and diapedesis (miR-874), cell cycle: G2/M DNA damage checkpoint regulation (miR-6524), retinol biosynthesis (miR-885) and TGF-β signalling (miR-2285t) (Table S9).

#### Enriched signalling pathways of target genes of differentially expressed miRNAs from lactation stage transition

About 39 (19 up- and 2 down-regulated), 61 (50 up- and 11 down-regulated) and 29 (18 up- and 11 down-regulated) highly significantly DE miRNAs (p-value < 8E-05 and |L2FC| > 2) between GAL and LAC, INV and GAL, and INV and LAC, respectively (Table S8d) were considered for target gene prediction and downstream analyses. For the comparison between INV and GAL, a lower threshold (p-value < 8E −05 and |L2FC|) > 1) was applied in order to keep important miRNAs for further analysis. A total of 4,030 and 4,956, 5,363 and 2,263, and 3,799 and 3,993 unique target genes were predicted with high confidence (experimentally validated or highly predicted) for up and down DE miRNAs between GAL and LAC, INV and GAL, and INV and LAC stages, respectively. The molecular and cellular processes and physiological system development functions enriched for each target gene group are shown in Table S10a and b.

**Figure 2:**
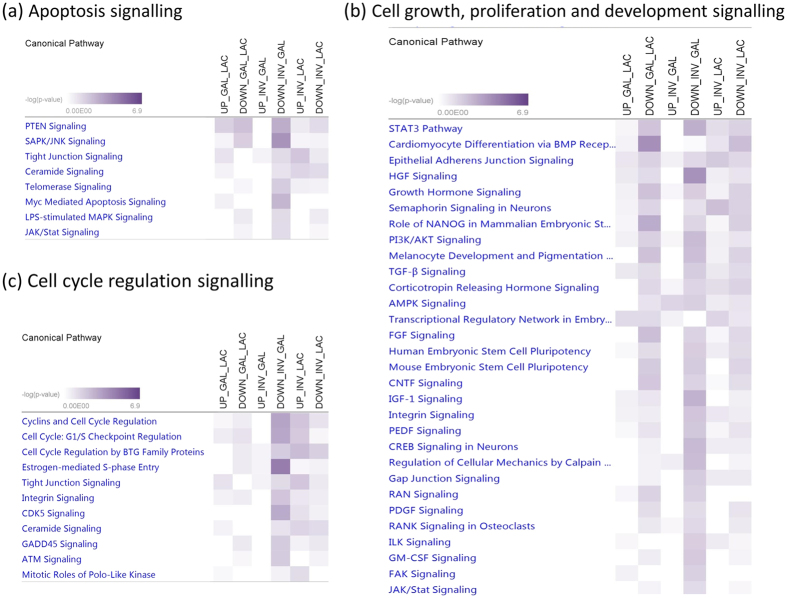
Signalling pathways enriched for target genes of differentially expressed miRNAs in each lactation transition stage: (**a**) apoptosis, (**b**) cell growth, proliferation and development and (**c**) cell cycle signalling. LAC: Lactogenesis; GAL: Galactopoiesis and INV: Involution.

**Figure 3:**
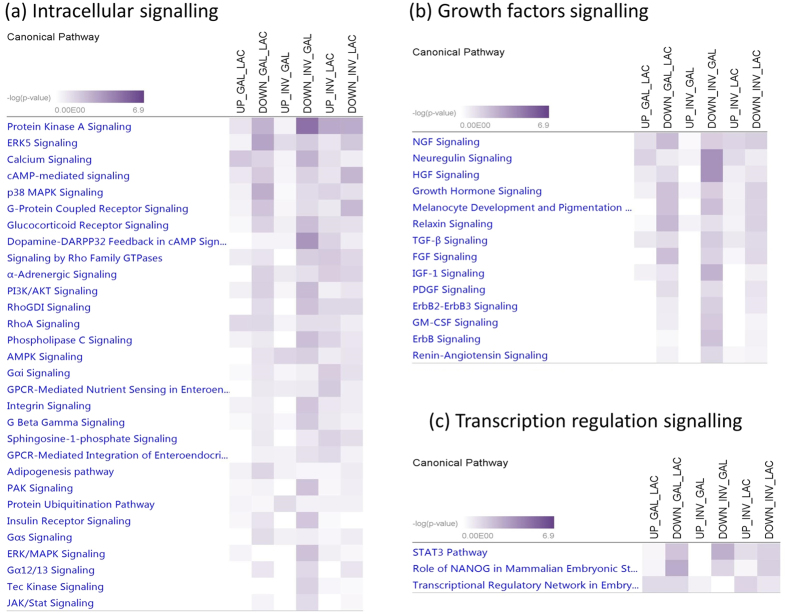
Signalling pathways enriched for target genes of differentially expressed miRNAs in each lactation transition stage: (**a**) intracellular, (**b**) growth factors and (**c**) transcription regulation signalling. LAC: Lactogenesis; GAL: Galactopoiesis and INV: Involution.

**Figure 4:**
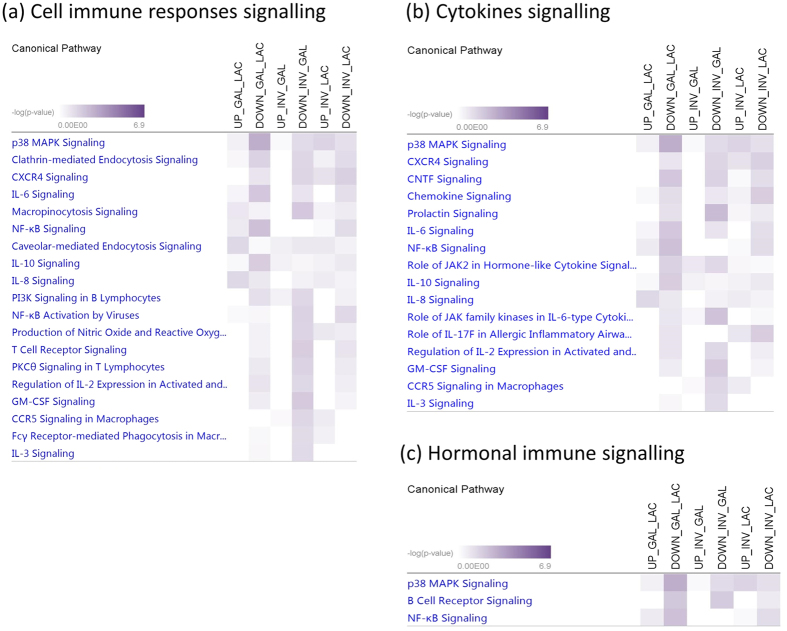
Signalling pathways enriched for target genes of differentially expressed miRNAs in each lactation transition stage: (**a**) cell immune responses, (**b**) cytokines and (**c**) hormonal immune signalling. LAC: Lactogenesis; GAL: Galactopoiesis and INV: Involution.

The number of pathways enriched for target genes of up and down DE miRNAs between GAL and LAC, INV and GAL, and INV and LAC were 12 and 70, 13 and 147, and 45 and 62, respectively (Table S11). Among these, enriched signalling pathways for apoptosis, cell cycle and cell growth, proliferation and development signalling are shown in Fig. 2a–c, intracellular and growth factor signalling are shown in Fig. 3a and b, and cell immune response, hormonal immune response and cytokines signalling are shown in Fig. 4a–c. In apoptosis signalling, the PTEN and tight junction signalling pathway was significantly enriched for target genes of both up and down DE miRNAs; meanwhile, LPS-stimulated MAPK signalling and JAK/Stat signalling were significantly enriched for only down DE miRNAs (Fig. 2a). In cell growth/proliferation and development category, STAT3 pathway and HGF signalling were significantly enriched for down DE miRNAs in all down comparisons (Fig. 2b). In cell cycle regulation signalling category, estrogen-mediated S-phase entry was the most significant pathway for down DE miRNAs (Fig. 2c). Protein Kinase A, ERK5, calcium, cAMP mediated- and p38 MAPK signalling were among the most significantly enriched pathways in the intracellular signalling category (Fig. 3a), meanwhile NGF, neuregulin, HGF and growth hormone signalling were important pathways for lactation in the group of growth factor signalling (Fig. 3b). Transcriptional regulatory network in embryonic stem cells, role of NANOG in mammalian embryonic stem cells and STAT3 pathway were three important transcriptional regulation signalling pathways enriched (Fig. 3c) for at least one comparison among lactation stages. The p38, MAPK, CXCR4, IL6, IL8, IL10 and NF-kβ signalling were significantly enriched in both categories involved in cell immune response and cytokines (Fig. 4a and b), and p38, MAPK and NF-kβ signalling were also important pathways enriched in hormonal immune signalling category (Fig. 4c). Prolactin was an important signalling pathway enriched for only down DE target gene groups (Fig. 4b).

#### Dynamically expressed miRNAs, their target genes and signalling pathways enrichment

**Figure 5:**
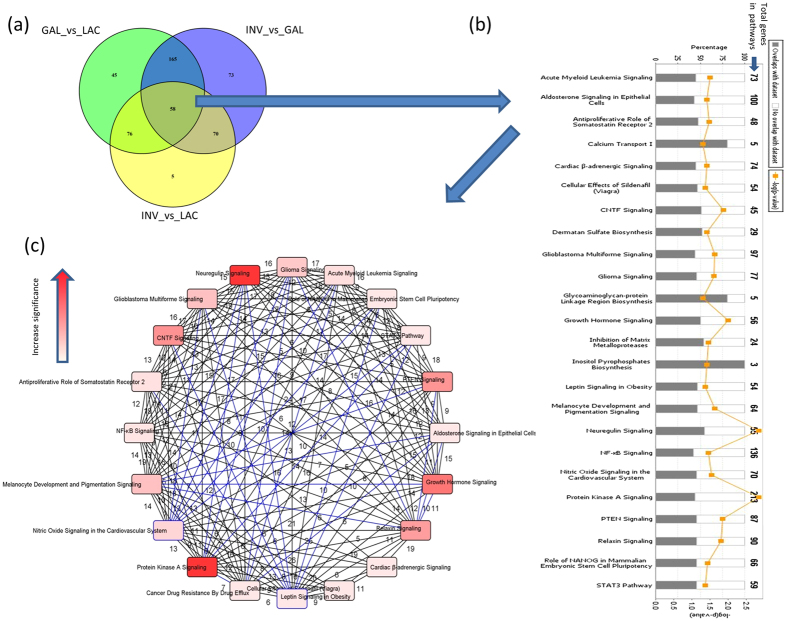
Enrichment results of miRNAs dynamically differentially expressed throughout the bovine lactation curve. (**a**) The Venn diagram indicates the number of dynamic miRNAs; (**b**) pathways enriched for target genes of dynamic miRNAs; and (**c**) circular overlap of common genes among significantly enriched pathways (outer squares, more intense red colour indicates higher degree of significance) for dynamic miRNA target genes. Numbers indicate the number of common genes between two pathways. LAC: Lactogenesis; GAL: Galactopoiesis and INV: Involution.

**Figure 6:**
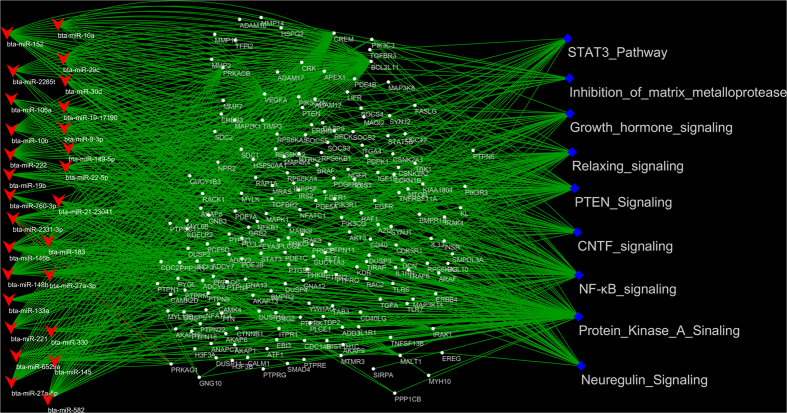
Mechanisms of dynamic differentially expressed miRNAs in significantly enriched pathways. To the left (red arrow head) are miRNAs which targets at least two genes (in the middle, white dots) in significantly enriched pathways (on the right, blue diamonds). Pathways enriched genes ranged from 2 to 94 and many genes were common to several pathways. Furthermore, several miRNAs targeted one or several genes.

A total of 58 miRNAs showed a dynamic DE pattern during the entire bovine lactation curve (Table 4), which altogether potentially target 5,491 unique genes. These target genes were significantly enriched in 24 canonical pathways (Fig. 5b and Table S12). The most important (significant) pathways included protein kinase A, neuregulin, growth hormone, CNTF, PTEN and relaxin signalling (Fig. 5b and c). However, several other relevant pathways for lactation were also enriched such as STAT3 pathway, leptin signalling in obesity and NF-κB signalling. These pathways shared many genes in common as shown in Fig. 5c. The inter-relationship between DE miRNAs and their important target genes and pathways is illustrated in Fig. 6. Moreover, 30 miRNAs among dynamic DE miRNAs were significantly correlated (Pearson correlation) with milk yield (p-value < 0.05). The expression of miR-6529a and miR-152 were the most significantly negatively (r = −0.47) and positively (r = 0.33) correlated with milk yield, respectively (Table 4).

#### Lactation stage specific differently expressed miRNAs, their target genes and signalling pathways enrichment

The number of lactation stage specific DE miRNAs were 28 (15 up- and 13 down-regulated), 53 (22 up- and 31 down- regulated) and 31 (10 up- and 21 down-regulated) for LAC, GAL and INV, respectively (Table S13). The most up- and down-regulated DE specific miRNAs were miR-2285ad (p-value = 8.29E-05) and miR-205 (p-value = 1.01E-11), miR-339b (p-value = 9.67E-04) and miR-EIA23-25909 (p-value = 3.82E-05), and miR-EIA20-21802 (p-value = 5.33E-04) and miR-2284j (p-value = 3.85E-06) for LAC, GAL and INV stages, respectively (Table 5). A total of 2,023 and 1,501, 3,059 and 3,817 and 2,716 and 1,869 unique genes were predicted to be potentially targeted by the up- and down-regulated specific DE miRNAs for LAC, GAL and INV stages, respectively. The signalling pathways enriched for target genes of stage specific miRNAs are shown in Figure S3.

### Time-course differential miRNA expression, target gene and signalling pathways enrichment

**Figure 7:**
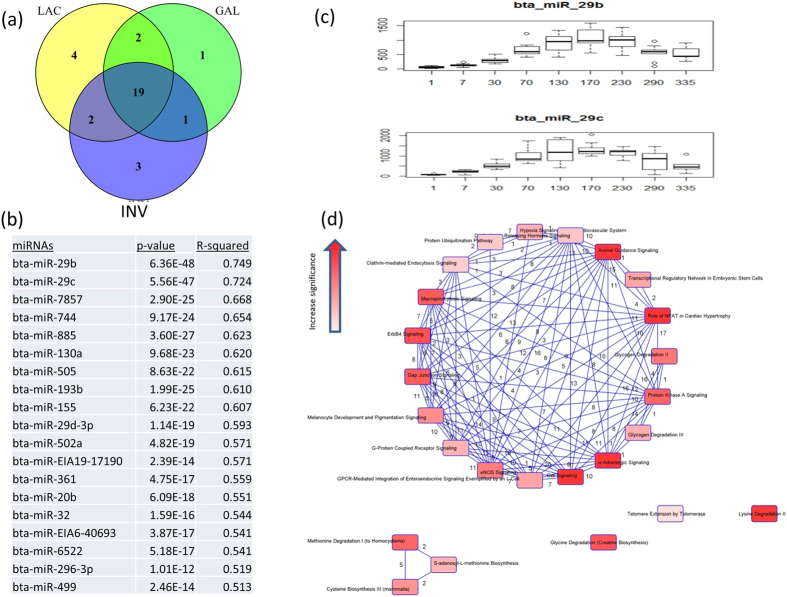
Enrichment results of time dependently expressed miRNAs throughout the bovine lactation curve. (**a**) The Venn diagram indicates the number of dynamic miRNAs (middle, 19 miRNAs); (**b**) 19 differentially expressed miRNAs, their p-values and r^2^ values reported from time course analysis, (**c**) expression pattern of two most important time dependent differentially expressed miRNAs (mir-29b and miR-29c), (**d**) circular overlap of common genes among significantly enriched pathways of dynamic miRNAs target genes. LAC: Lactogenesis; GAL: Galactopoiesis and INV: Involution.

To identify miRNAs with significant temporal expression patterns and significant differences between lactation stages, we performed a time-course expression analysis according to lactation day. The number of miRNAs significantly DE by time were 27, 23 and 25 in LAC, GAL and INV respectively. Among them, 19 were common to all stages (Fig. 7a). The highest significantly DE miRNAs in a time course manner were two members of miR-29 family: miR-29b (p-value = 6.36E-48, r^2^ = 0.749) and miR-29c (p-value = 5.56E- 48, r^2^ = 0.724) (Fig. 7b). The two miRNAs had similar expression patterns that increased with increasing days in lactation, reaching high levels by D130 to D230, followed by a rapid decline until end of lactation (Fig. 7c). The 19 common miRNAs were predicted to target 1,843 genes which were significantly enriched in 37 canonical pathways (Fig. 7d and Table S14). Gαi signalling and α-adrenergic signalling were the most significantly enriched pathways meanwhile melanocyte development and pigmentation signalling and protein kinase A signalling were the only two common pathways enriched for dynamic DE and time course DE target genes.

### Real-time quantitative PCR validation of identified miRNA expression profiles

**Figure 8:**
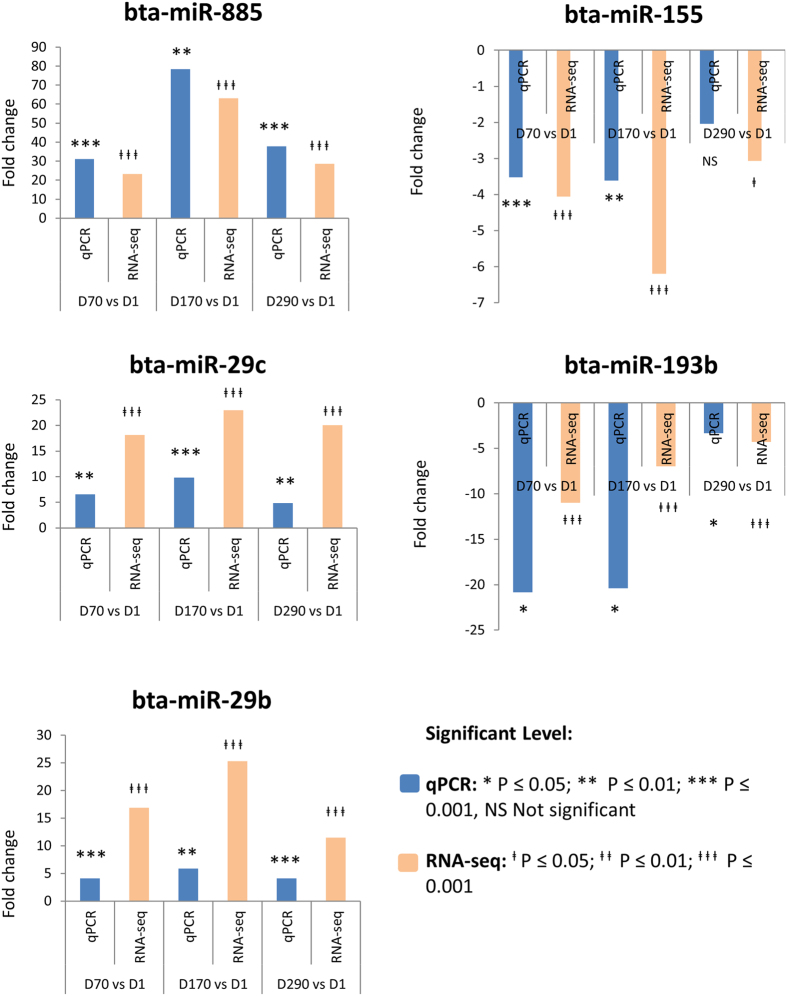
Result of qPCR validation of the expression of five miRNAs on day 1, 70, 170 and 290 and compared to miRNA-Seq results.

Real-time quantitative PCR (qPCR) was used to validate the miRNA-Sequencing expression results of selected DE miRNAs identified in this study (Fig. 8). Expression trends of selected miRNAs by qPCR were generally similar to the results from miRNA-sequencing. For instance, miR-885, miR-29b and miR-29c showed a significantly higher expression at D70, D170 and D290 compared with D1 (p < 0.05) after RNA-sequencing and confirmed by qPCR meanwhile miR-193b and miR-155 showed a significantly lower expression at D70, D170 and D290 compared with D1 (p < 0.05) after RNA-sequencing and confirmed by qPCR (Fig. 8).

## Discussion

Lactation, a dynamic process that involves mammary gland development, synthesis and secretion of milk is among the most remarkable products of evolution. This study investigated the roles of miRNAs in this dynamic process throughout the bovine lactation curve. Particularly, we examined how miRNA regulate/mediate the changes in the lactation curve from lactogenesis to galactopoiesis and from galactopoiesis to involution.

The method of deep miRNA-sequencing was used to detect expressed miRNAs at the different stages of the bovine lactation curve. A total of 475 known miRNAs were detected, corresponding to over 60% of known bovine miRNAs in miRBase Release 21. In previous studies, 417, 321, and 310 known miRNAs from 8, 36 and 4 small RNA libraries derived from milk exosomes^7^, lactating mammary gland tissue^26^, and milk fat^23^, respectively have been reported. Despite these differences, the number of highly expressed miRNAs in our study is mostly in agreement with these studies (Table 1). These highly, commonly expressed miRNAs suggest their potential regulatory functions in mammary gland development and productivity. This notion is supported by biological process and pathway enrichment results since many basic cellular and molecular processes^27^,^28^ and immunlogical pathways were enriched for these commonly highly expressed miRNAs (Table 2).

Moreover, we also detected 238 novel miRNAs in this study. Interestingly, the novel miR-EIA3-33361 was expressed at appreciable levels throughout the lactation curve and may play similar roles as for known highly expressed miRNAs described above. Target gene enrichement for this miRNA indicated that it may regulate basic cellular processes and also have roles in immunlogical, and hormone pathways. For instance, the estrogene receptor signalling pathway was the most significantly enriched and is important for mammary gland development and for lactation regualtation^29^,^30^,^31^. Nevertheless, the detection of novel miRNAs is the first step to further exploration of their functions. The novel miRNAs identified in this study will enrich the bovine miRNome repertoire and contribute to better understanding of the biology of milk secretion and mammary gland development.

From a physiological perspective, major metabolic changes take place at the initiation of lactation to accommodate increased demand for nutrients and energy to support milk synthesis. MiRNAs might play important regulatory roles to balance activities during lactation by modulating the expression of genes in specific pathways. In this study, we grouped the examined days into three stages according to physiology of the lactation curve (Figure S2), principle component analysis and between lactation days DE results (Fig. 1). Therefore, we performed a comparison of miRNA expression between the three stages of lactation (LAC, GAL and INV) to detect important miRNAs and their possible regulatory mechanisms during each physiological stage and also at the transition points.

Interestingly, the expression of more than half the miRNAs in this study changed significantly from LAC to GAL stage (p-value < 0.05) (Table S8). Notably, several of the significant DE miRNAs (miR-29a, miR-29b, miR-29c, miR-29d-3p, miR-885, miR-490, miR-146b and miR-363) have been reported to play roles in mammogenesis, LAC and GAL^4^,^32^. Particularly, many members of miR-29 family were up-regulated in GAL compared to LAC stage. The miR-29 family have been reported as important regulators of many cellular mechanisms of cancer and human diseases (reviewed in refs 33, 34, 35, 36). Interestingly, miR-29b was differentially expressed between colostrum and mature milk^32^. Notably, many target genes of miR-29b were enriched in PTEN, PI3K/AKT and JAK/Stat signalling pathways, which are essential for prolactin hormone function in lactation, and triacylglycerol biosynthesis pathway.

Little is known about the function of miR-363, the top most down-regulated miRNA when switching from LAC to GAL in lactation, except that it has been reported to be significantly DE between lactating and non-lactating cows^4^. The target genes of these miRNAs were enriched in many pathways of relevance in the lactation process^37^,^38^ (Table S9). The most up-regulated miRNA between GAL and INV stages was miR-874 (p-value = 9.37E-14). Its target genes were enriched for lipid related pathways (phospholipases and eicosanoid signalling) (Table S11). MiR-874 potentially targets *CLDN10, RAB13* and *CLDN18* in tight junctions signalling pathway. The role of tight junctions during mammary development and lactation have been reviewed in several studies^37^,^38^. MiR-6524 (p-value = 1.84E-09) was the most down-regulated miRNA between GAL and INV and it can target *YWHAQ, RPS6KB1* and *PTPN11* in ERK5 and IGF1 signalling pathways or *RPS6KB1* and *PTPN11* in CNTF signalling pathway. The IGF system was shown to inhibit the involution of the mammary gland in mice^39^ while CNTF was shown to activate the STAT3 pathway in rats/neuroblastoma cells^40^,^41^, therefore miR-6524 can indirectly influence lactation via the STAT3 pathway.

MiR-885 was the most up-regulated miRNA when GAL was compared with LAC and when INV was compared with GAL (Table S8). This suggests an important role for miR-885 during the entire lactation curve. MiR-885 can target *RET* and *FRS2* genes in the glial-derived neurotrophic factor (GDNF) family ligand-receptor interactions pathway or *DAPK1, CDK6* and *NEK2* genes in pyridoxal 5′-phosphate salvage pathway. Since the GDNF-family ligands are crucial for the development and maintenance of distinct sets of central and peripheral neurons^42^ as well as maintain dopamine neurons and moto neurons in the centre nervous system^43^, it possibly regulate changes in lactation activities via the activities of dopamine. MiR-2285t (p-value = 7.18E −24), the most down-regulated miRNA between INV and GAL stages can target *TGFBR1, RRAS2, RNF111, BMP2* and *ACVR2A* molecules in TGF-β signalling pathway. The TGF-β pathway is known to significantly regulate mammary development^44^ and different isoforms of TGF-β are expressed in distinct spatial and temporal patterns^44^,^45^,^46^.

The switch between lactation stages is accompanied by changes in alveolar cell number, milk secretion activities and milk removal. As expected, many pathways related to apoptosis as well as cellular development were enriched (Fig. 2). For instance, PTEN signalling and STAT3 were the top pathways enriched among these comparisons (Fig. 4). The *PTEN* gene is an important target for miR-29b in the regulation of mammary gland development^47^. PTEN signalling is crucial for the activities of prolactin autocrine^48^. The initiation of lactation is known to require induction of autocrine prolactin, and the level of this autocrine is known to be endogenously regulated by the signal of PTEN-PI3K-AKT pathway^48^. The STAT3 pathway, which is crucial for apoptosis and mammary gland development^49^, was significantly enriched for target genes of down-regulated DE miRNAs, an indication of the release of miRNA inhibition and consequently an enhancement of pathway gene expression and activities at the end of the lactation curve. Estrogen regulates the development of the mammary gland and induction of lactation^49^,^50^, and it is an important hormone for the onset of lactation^51^. The changes in this pathway between GAL and INV might be due to the role of estrogen in pregnancy. Athie *et al*.^52^ indicated that exogenous estrogen accelerates mammary involution at the end of lactation.

MiRNAs are not only important for the regulation of apoptosis and cellular development, but also for the regulation of intracellular signalling. Mitogen-activated protein kinase signalling (MAPK) is one of the key signalling nodes in mammary gland development^53^. Extracellular-signal-regulated kinase 5 (ERK5) is a member of the MAPK, and ERK5 together with protein kinase A signalling pathways were the most significantly enriched pathways for down-regulated miRNA target genes for transition from LAC to GAL (Fig. 3a). Recently, *ERK5* was showed to mediate the prolactin signal^54^ via regulation of dopamine transport^55^. Protein kinase-A is an enzyme that regulates diverse processes including cellular growth, and development and metabolism^56^. Lactation process is regulated by circulating reproductive and metabolic hormones and the roles of these hormones during lactating as well as in the whole lactation process have been reviewed by several authors^31^,^51^,^57^. Therefore, it is not surprising that prolactin signalling was among the important pathways enriched for target genes of DE miRNAs in this study (Fig. 4b). Similarly, growth hormone and IGF-1 signalling were also enriched for target genes of down-regulated DE miRNAs between LAC and INV stages. The importance of these hormones in the lactation process is well documented^31^,^51^,^57^. A number of immunological pathways (such as IL-8, IL-6, IL-10, NF-kβ signalling) were significantly enriched in this study (Fig. 4a) therefore indicating the importance of immune related genes and pathways for milk production. The immune mechanisms are important to protect cell secreting organs and also the offspring^58^. MiRNAs with specific immune functions in lactogenesis, also called colostrum milk have been reported by many authors (see review ref. 58). Moreover, a higher number of immunological pathways were enriched for target genes of down-regulated miRNAs thus suggesting that miRNAs may play roles in the up-regulation of these pathways during lactation.

To further explore miRNA roles in the regulation of the lactation curve, we also examined the miRNAs dynamically DE when comparing expression of miRNAs between lactation stages. MiR-2285t showed the most dynamic expression pattern (Table 4) with significantly decreased expression after onset of lactation. MiR-2285t was also significantly correlated with milk yield (Table 4). The roles of miR-2285t have been discussed above. The functions of several dynamic DE miRNAs with significant correlations with milk yield in this study (Table 4) have been validated in ruminant mammary gland cells. It was shown that miR-145 regulate lipogenesis via targeting the *INSIG1* gene^59^; miR-27a suppress triglyceride accumulation via targeting *PPARɣ* gene^60^ while miR-152 enhance viability and multiplication capacity of cow mammary gland cells through regulation of *DNMT1* gene^10^. However, both the most positively and negatively correlated miRNAs (miR-EIA8-45133 and miR-6529a) with milk yield have not been functionally validated in ruminants. Nevertheless, since milk yield is under the control of many factors, the simple correlations between miRNA and milk yield reported in this study might not adequately reflect the roles of these miRNAs in milk production. Hence, functional validation of the roles of these miRNAs in milk production is required. In fact, many pathways enriched for dynamic DE miRNAs were also enriched for at least one lactation switch point (e.g. STAT3 pathway, PTEN and growth hormone signalling, relaxing signalling, CNTF signalling, NF-k β signalling, neuregulin signalling and protein kinase A signalling), except for inhibition of matrix metalloproteinase pathway (Fig. 6). The matrix metalloproteinases (MMPs) play key roles in remodelling extracellular matrix^61^ which is an important regulator of mammary epithelial cell function essential for cell proliferation, differentiation, and survival in the mammary gland. The dynamic miRNAs can target 13 (*HSPG2, TIMP3, ADAM17, MMP7, SDC1, RECK, MMP16, MMP14, MMP2, TFPI2, ADAM12, SDC2, ADAM10*) out of 24 genes (Table S12, Fig. 5b) in the inhibition of the matrix metalloproteinase pathway indicating that these miRNAs are very important for inactivation of MMPs.

Besides exploring the dynamic DE miRNAs, we also reported significant DE miRNAs that were specific to each lactation stage (Table 5 and S13). MiR-205, the most specific DE miRNA in LAC stage (Table 5) is known to regulate epithelial to mesenchymal transition^62^. It was also reported to be highly up-regulated at day 30 postpartum compared to day-30 prepartum in cows^63^ but not significantly DE between day 7 and day 30 postpartum. Meanwhile the most significant specific DE miRNAs for GAL (miR-EIA23-25909) and INV (miR-2284j) stages have not been functionally validated in dairy mammary gland. Enrichments for target genes of stage specific DE miRNAs (Figure S3) showed importance of PTEN signalling pathway for LAC^48^, prolactin signalling pathway for GAL and INV^64^ and STAT3 pathway for INV stages^65^.

To provide a comprehensive understanding of lactation regulation, it is also necessary to explore lactation as a time dependent process in which, miRNAs might act as time-dependent regulators. However, analysis of time-series miRNA-Seq data is immature in terms of method development^66^. Here we adapted the Next maSigPro package which is used for the identification of DE genes from time-course mRNA-Seq data to our miRNA sequencing data. The package efficiently controls both false-positive and false-negative detection rates^67^. A total of 19 miRNAs, 17 known and two novel (miR-EIA19-17190 and miR-EIA6-40693), were commonly expressed among the three lactation stages (Fig. 7a). Interestingly, these two novel miRNAs were also dynamically DE expressed. MiR-29b and miR-29c were highly significantly DE by time (p-value = 6.35E-48 and 5.56E-47, respectively) and are the most interesting miRNAs detected from time course analysis. Notably, miR-29b was not reported as a dynamic miRNA since it was not significantly DE between INV and GAL stages (p-value BH = 0.06). The potential functions of miR-29c in mammary gland and lactation have been suggested in several studies^68^,^69^,^70^. Significantly DE miRNAs from time course analysis (Next maSigPro package) and DE miRNAs using Deseq2 package have relatively small overlap (5 miRNAs) which could be due to the differences in the manner in which the softwares treat data. Fewer significantly DE miRNAs (19 miRNAs) obtained from the Next maSigPro were expected since many of them were removed in the second step. Even at that, a relatively high number of pathways (37) were enriched for the target genes of these miRNAs, with only two overlap (protein kinase A signalling and melanocyte development and pigmentation signalling) with pathways enriched for dynamic DE miRNAs. Roles for protein kinase A signalling have been described above but it is not clear how melanocyte development and pigmentation signalling pathway is linked to lactation. The most significantly enriched pathway was α-adrenergic signalling. This pathway acts via α-adrenergic receptors which serve critical roles in maintaining homeostasis in normal physiologic settings^71^. Some relevant pathways enriched for time course miRNAs were corticotrophin releasing hormone signalling, gap junction signalling, G-protein coupled receptor signalling, ERK/MAPK signalling, ErbB4 signalling and CDK5 signalling which have been reported to be directly involved in lactation processes^72^,^73^.

It is important to note that some decisions or softwares used might have influenced the outcome of our results. Firstly, since many miRNAs were DE in this study, we used stringent thresholds to prioritise DE miRNAs for downstream analyses and by so doing risk losing important miRNAs that did not pass set thresholds and consequently valuable information. Secondly, although we have demonstrated that milk fat from mid lactation cows can serve as a source of RNA for the study of mammary gland biology^23^, our study did not compare other stages of lactation. However, other authors have confirmed that milk can serve as a source of RNA for the study of mammary gland functions^74^,^75^,^76^,^77^. In fact, it has been indicated that human milk fat transcriptome contained genes uniquely expressed in lactating mammary epithelial cells^78^. Furthermore, obtaining mammary gland biopsy on the same animal nine times during a lactation circle would have had negative effects on animal health and productivity. Thirdly, target genes were purely based on predictions as well as from experimental observations in biological systems other than bovine (from IPA software) so the functions of these miRNAs in bovine needs to be validated.

In conclusion, the miRNome of milk fat was characterized during the entire bovine lactation curve. Novel miRNAs detected will enrich the bovine miRNome repertoire. Several miRNAs were important (potential) regulators at each lactation stage switch: miR-29b and miR-363 for LAC to GAL switch, and miR-874 and miR-6254 for GAL to INV switch. Moreover, we also reported specific DE miRNAs for each lactation stage. Several signalling pathways important for the lactation process (protein kinase A, prolactin, PTEN, TGF-β, ERK5 and STAT3 pathways) were enriched for target genes of significantly DE miRNAs and may be central pathways for regulatory mechanisms of the bovine lactation curve. MiRNAs that had both dynamic and time dependent expression patterns during the lactation curve (e.g. miR-29c, miR-EIA19-17190 and miR-EIA6-40693) were revealed. Overall, our data suggest that miRNAs serves diverse, temporal, physiological signal–dependent regulatory functions during lactation. Further studies are required to specify the roles of important miRNAs detected in this study in lactation-relevant pathways as well as their roles in crosstalk among pathways.

## Materials and Methods

### Animal management and milk sampling

Animal use and experimental procedures were according to the national codes of practice for the care and handling of farm animals (http://www.nfacc.ca/codes-of-practice) and approved by the Animal Care and Ethics Committee of Agriculture and Agri-Food Canada.

Nine healthy Canadian Holstein cows, first to third parity, were used. Cows were fed a mixed ration of corn and grass silages (50:50) and concentrate and managed following standard procedures. Animals were housed in individual tie stalls and allowed *ad libitum* access to feed and water at all times. Daily milk yield for each cow was recorded with electronic milk meters (MU-480, De Laval Inc., USA) and the comparison of milk yield between lactation days is shown in Figure S2. The shape of the lactation curve was calculated according to Wood’s model^24^ (using an incomplete gamma distribution) (Figure S2). Milk samples for RNA isolation were collected from all cows at different times throughout the lactation curve as follows: lactogenesis (**LAC**) (D1 [day of calving] and D7), galactopoiesis (**GAL**) (D30, D70, D130, D170 and D230), and involution (**INV**) (D290 and when milk production had dropped to 5 kg/day (D5kg) (drying off period, ~day 335)^20^,^21^,^22^. A volume of 50 mL fresh milk was collected from the back quarters (25 ml/back quarter) of each cow three hours after the morning milking and immediately placed on ice. Samples were transferred to the laboratory and immediately processed to reduce potential RNA degradation. In the laboratory, milk was mixed well and centrifuged at 1,900 g for 15 min. The fat layer (upper phase) was transferred to a 50 mL RNase free falcon tube and ~7.5 mL Qiazol lysis reagent (Qiagen Inc., Canada) was added, vigorously mixed by vortexing until the fat was well dispersed. The homogenized fat was stored at −80 °C until used.

### Total RNA extraction

Total RNA was extracted from fat homogenate using miRVana miRNA isolation kit (Life Technologies, USA) according to manufacturer’s instructions. Briefly, 5 mL fat homogenate was thawed in a water bath (37 °C) for 5 min followed by incubation at room temperature for 5 min. The tube was vortexed briefly and 1.8 mL fat homogenate was aliquoted into each of 3 × 2 mL tubes followed by centrifugation at 12,000 × g, 4 °C for 10 min under a fume hood. From each tube, 800 μL lysate below the fat layer was transferred to a new 2 mL tube, 80 μL (1/10 volume) of miRNA Homogenate additive was added, mixed well by vortexing or inverting several times followed by incubation on ice for 10 min. To separate the aqueous and organic phases, 800 μL acid-phenol:chloroform was added, mixed by vortexing for 30–60 sec followed by centrifugation for 10 min at 12,000 × g (4 °C). About 450 μL of the upper aqueous phase was carefully transferred into a 1.5 mL tube, 562.5 μL 100% ethanol was added and mixed thoroughly by inverting several times. Up to 700 μL (at a time for greater volumes) of this lysate/ethanol mixture was transferred to a filter cartridge and centrifuged for 15 sec at 10,000 × g at 4 °C. The flow through was discarded and washed with miRNA wash solution (700 μL at first wash and 500 μL at second and third washes) each time centrifuging for 15 sec at 10,000 × g at 4 °C and discarding the flow through. Total RNA was eluted by adding 100 μl Elution Solution or Nuclease-free Water (heated to 95 °C) followed by centrifugation for 1 min at 10,000 × g at 4 °C. Total RNA was digested with Turbo DNAse (Ambion Inc., USA) to remove genomic DNA contaminant followed by purification with Zymo RNA clean & concentrator-25 kit (Zymo Research, USA). The quantity of isolated RNA was measured with NanoDrop 1000 (NanoDrop Technologies, USA) while RNA integrity was determined on an Agilent 2100 Bioanalyzer using an RNA 6000 Pico kit (both from Agilent Technologies, USA) (Figure S2).

### miRNA library preparation and sequencing

A total of 71 libraries were prepared and barcoded for sequencing according to a previous protocol^79^ with minor modifications. Briefly, total RNA was first ligated to a primer at the 3′end (3′adaptor) by T4 RNA Ligase 22tr K227Q (New England Biolabs Inc., Canada) which was then annealed to a reverse transcription primer to prevent undesirable dimerization of 3′ and 5′ adaptors in the following step. Before reverse transcription, the 5′adaptor was ligated to the 5′end of the RNA by T4 RNA Ligase 1 (New England Biolabs Inc., Canada). This RNA:DNA hybrid was reverse transcribed into cDNA using Superscript III kit (Life Technologies, USA). The barcoding of the libraries was achieved by PCR using primers which consisted of specific barcodes for each library. Size separation of the miRNA libraries was performed by polyacrylamide gel electrophoresis. The libraries were then eluted from the gel using an elution buffer (10 mM Tris-Hcl pH 7.5; 50 mM NaCl, 1 mM EDTA) and concentrated using DNA clean and concentrator-5 (Zymo Research, USA). Finally, the concentration of the purified libraries was evaluated by Picogreen assay (Life Technologies, USA) on a Nanodrop 3300 fluorescent spectrophotometer. Multiplexed libraries (18 libraries per lane) were subjected to 50 bp single end sequencing on an Illumina HiSeq 2000 system (Illumina Inc., USA) by McGill University and Genome Quebec Innovation Centre (http://www.gqinnovationcenter.com/index.aspx) using TruSeq v3 (Illumina Inc., USA) reagents. The raw fastq files of the sequence data have been submitted to NCBI Sequence Read Archive database with accession number SRP081157 and novel miRNA sequences have been submitted to miRBase.

### Small RNA sequence data analysis

The raw data of 71 fastaq files were checked for sequencing quality with FastQC program version 0.11.3 (http://www.bioinformatics.babraham.ac.uk/projects/fastqc/). Cutadapt v1.2.2 (http://code.google.com/p/cutadapt/) was used to trim 3′ adaptor sequences and filter 5′ adaptor contaminants and repeats. Reads shorter than 18 nucleotides after trimming or having a low Phred score of less than 20 for at least 50% of the bases were discarded. Low quality bases (Phred score < 20) as well as reads containing unknown bases were removed using FASTQ Quality Filter tool of FASTX-toolkit (http://hannonlab.cshl.edu/fastx_toolkit/). For novel miRNA discovery, clean reads from the 71 files that passed all filtering criteria were parsed into one file and mapped to the bovine genome (UMD3.1) using bowtie 1.0.0^80^. Reads that mapped to more than five positions of the genome were discarded. Furthermore, reads that mapped to bovine rRNA, tRNA, snRNA and snoRNA in the Rfam RNA family database (http://rfam.sanger.ac.uk/) were removed.

### Identification of known miRNA and novel miRNA discovery

The identification of known miRNA and discovery of novel miRNA were performed using miRBase v21 and miRDeep2 v2.0.0.7^81^, respectively. MiRDeep2 uses a probabilistic algorithm based on the miRNA biogenesis model and designed to detect miRNAs from deep sequencing reads. The core and Quantifier modules of miRDeep2 were used to discover novel miRNAs in the pooled dataset of all the libraries. The Quantifier module of miRDeep2 was used to profile the detected miRNAs in each library. In order to predict novel miRNAs with high confidence, only those with a miRDeep2 score higher than five were considered as novel miRNAs. Subsequent threshold of 10 counts per million and present in at least 7 libraries was used for both known and novel miRNAs. Only miRNAs meeting these criteria were retained for differential expression analysis.

### Differential miRNA expression analysis

The R (v3.1.3) package Deseq2 (v1.11.19)^82^ which uses a negative binomial model was used to identify significantly DE miRNAs throughout lactation and between the 3 stages of lactation (LAC, GAL, INV). To find stage specific DE miRNAs, additional analyses were performed to compare each lactation stage with the two other stages. Significantly DE miRNAs were defined as having a Benjamini and Hochberg^25^ corrected p-value (p-value BH) ≤ 0.05. The miRNAs with the adapted BH threshold (p-value BH < 8e-05 and |L2FC| > 2) were considered as highly significantly DE and were used for downstream and pathway analyses. The choice of this threshold was to enable selection of more relevant miRNAs in the downstream enrichment analyses.

### Time course analysis

The R package, Next maSigPro^67^ was used for time course analysis of expression data. We adapted a two-step regression strategy to find miRNAs with significant temporal expression changes and significant differences between lactation stages. Firstly, we performed a quadric polynomial regression model using p.vector() function. The significantly DE miRNAs from this regression model were obtained by applying a FDR of 5% (Q = 0.05). Secondly, a variable selection procedure employed a stepwise regression (step.method= “two.ways.backward”, alfa = 0.05) was applied by using T.fit() function). The lists of DE miRNAs were extracted (using get.siggenes() function) for each lactation stage with r^2^ ≥ 0.5^67^.

### Function analyses of significantly differentially expressed miRNAs

The target genes of DE miRNAs were predicted using Ingenuity Pathway Analysis (IPA) software (Ingenuity Systems Inc., USA, http://www.ingenuity.com/products/ipa). The miRNA targets were filtered based on experimentally observed or predicted with high confidence. Moreover, for miRNAs without targets in IPA database, the perl script from the TargetScan website (http://targetscan.org) was used to predict (targetscan_60.pl) and to calculate the context scores (targetscan_61_context_scores.pl) of their gene targets. Predicted targets with context scores above 95th percentile were further used^26^. Function analysis of miRNA target genes was performed using IPA core analysis function. The core analysis identifies statistically significant over-representation of predicted target genes in a given biological process and pathway. Biological processes and pathways with Benjamini and Hochberg^25^ corrected p-values ≤ 0.05 and contained at least 2 target genes from our data were considered to be significantly over-represented in our samples.

### Real time quantitative PCR (qPCR)

Total RNA (10 ng each) from the same samples used in miRNA sequencing and collected on D1, D70, D170 and D280 were reverse transcribed using Universal cDNA Synthesis Kit II from Exiqon (Exiqon Inc., USA). The cDNA was then diluted 1:80 in nuclease-free water and subjected to quantitative qPCR on a Stepone Plus System (Applied Biosystems, USA) using an ExiLENT SYBR^®^ Green Master Mix Kit (Exiqon, USA) and the miRCURY LNA™ Assay (Exiqon, USA) according to the manufacturer’s instructions. The comparative Ct (ΔΔCt) method was used to determine the expression level of miRNA. The geometric mean of miR-103 and miR-25 was used as endogenous control.

## Additional Information

**How to cite this article:** Do, D. N. *et al*. MicroRNA roles in signaling during lactation: an insight from differential expression, time course and pathway analyses of deep sequence data. *Sci. Rep.* **7**, 44605; doi: 10.1038/srep44605 (2017).

**Publisher's note:** Springer Nature remains neutral with regard to jurisdictional claims in published maps and institutional affiliations.

## Supplementary Material

Supplementary Information

Supplementary Table S1

Supplementary Table S2

Supplementary Table S3

Supplementary Table S4

Supplementary Table S5

Supplementary Table S6

Supplementary Table S7

Supplementary Table S8

Supplementary Table S9

Supplementary Table S10

Supplementary Table S11

Supplementary Table S12

Supplementary Table S14

## Figures and Tables

**Table 1:** Abundantly expressed miRNAs across lactation stages and the consistency in their expression levels with previous studies on bovine milk and mammary gland tissues/epithelial cells.

miRNA	Lactogenesis		Galactopoiesis		Involution		Recently reported as highly expressed
	Total Read	%	Total Read	%	Total Read	%	
bta-miR-30a-5p	5306615	13.84	13904630	13.18	2259298	6.65	4,23,83, 84, 85
bta-miR-21-5p	4649150	12.12	8916972	8.45	6116880	17.99	4,23,83, 84, 85
**bta-miR-26a**	**4069639**	**10.61**	**10993251**	**10.42**	**4617897**	**13.58**	4,23,83, 84, 85
**bta-miR-148a**	**4055500**	**10.57**	**17599046**	**16.69**	**3649411**	**10.73**	4,23,83, 84, 85
bta-let-7a-5p	2977450	7.76	6863855	6.51	2395954	7.05	4,23,83, 84, 85
bta-let-7b	1515893	3.95	4326764	4.10	1043005	3.07	4,23,83, 84, 85
bta-miR-99a-5p	1378941	3.60	4441195	4.21	1056461	3.11	4,23,83, 84, 85
bta-miR-191	1053447	2.75	2045763	1.94	1787691	5.26	4,23,83, 84, 85
bta-let-7f	866825	2.26	1478554	1.40	906602	2.67	4,84,85
bta-miR-30d	822892	2.15	3266417	3.10	1193706	3.51	23,83, 84, 85
bta-miR-200c	677105	1.77	3738531	3.54	884574	2.60	4,23,83, 84, 85
bta-miR-200a	619618	1.62	3204582	3.04	598686	1.76	4,23,83, 84, 85
bta-miR-186	582128	1.52	2041651	1.94	637989	1.88	4,23,83, 84, 85
bta-let-7g	501386	1.31	1072697	1.02	459179	1.35	4,23,83
bta-miR-92a	498594	1.30	1346696	1.28	569334	1.67	4,23,83, 84, 85

**Table 2:** Significantly enriched biological functions by the target genes (5,644) of 15 highly expressed miRNAs across lactation stages.

Category	p-value BH	Category	p-value BH
Molecular and Cellular Functions		Physiological system development and function	
Gene expression	6.06E-20 to 8.38E-06	Organismal survival	2.69E-18 to 7.49E-13
Cell morphology	4.26E-14 to 6.09E-05	Embryonic development	6.18E-18 to 5.84E-05
Cellular assembly and organization	6E-14 to 5.84E-05	Organismal development	6.18E-18 to 6.86E-05
Cellular function and maintenance	6E-14 to 5.84E-05	Nervous system development and function	6.63E-18 to 6.93E-05
Cellular development	2.28E-13 to 5.53E-05	Organ development	3.77E-15 to 5.84E-05
Cellular growth and proliferation	2.28E-13 to 6.19E-05	Tissue development	3.77E-15 to 6.57E-05
Cellular movement	9.33E-12 to 6.93E-05	Cardiovascular system development and function	5.79E-15 to 6.57E-05
Cell death and survival	2.81E-09 to 6.77E-05	Behavior	1.65E-13 to 1.78E-05
Protein synthesis	4.45E-08 to 4.7E-07	Tissue morphology	1.43E-12 to 6.77E-05
Amino acid metabolism	5.24E-08 to 2.7E-05	Skeletal and muscular system development and function	2.25E-12 to 6.86E-05
Post-translational modification	5.24E-08 to 5.23E-05	Digestive system development and function	5.33E-12 to 4.58E-06
Small molecule biochemistry	5.24E-08 to 4.85E-05	Reproductive system development and function	1.4E-10 to 5.84E-05
Cell cycle	7.19E-08 to 3.37E-05	Hair and skin development and function	4.18E-10 to 3.93E-05
Carbohydrate metabolism	2.4E-07 to 4.85E-05	Organ morphology	6.05E-10 to 6.42E-05
Molecular transport	2.4E-07 to 5.29E-07	Connective tissue development and function	3.23E-08 to 3.93E-05
Cell signalling	4.7E-07 to 5.84E-05	Hematological system development and function	3.23E-08 to 2.78E-05
Cell-to-cell signalling and interaction	7.58E-07 to 5.84E-05	Hematopoiesis	3.23E-08 to 2.78E-05
Cellular compromise	2.58E-06 to 2.58E-06	Respiratory system development and function	4.11E-08 to 9.76E-06
Energy production	3.95E-05 to 3.95E-05	Visual system development and function	3.48E-06 to 3.48E-06
Lipid metabolism	3.95E-05 to 3.95E-05	Hepatic system development and function	4.58E-06 to 4.58E-06
—	—	Auditory and vestibular system development and function	4.72E-06 to 4.72E-06
—	—	Renal and urological system development and function	6.09E-05 to 6.09E-05

**Table 3:** Top* most significantly differentially expressed miRNAs in each lactation transition stage.

miRNAs	L2FC^2^	p-value BH	miRNAs	L2FC^2^	p-value BH
**Galactopoiesis and lactogenesis**			bta-miR-2344	4.19	8.53E-06
bta-miR-29b	3.02	2.99E-31	bta-miR-EIA19-19133	4.03	2.54E-09
bta-miR-885	3.97	3.21E-29	bta-miR-EIA21-23794	3.98	2.00E-06
bta-miR-29c	2.77	2.34E-26	bta-miR-EIAX-48475	3.88	9.12E-06
bta-miR-29a	1.46	6.18E-24	bta-miR-223	3.80	1.53E-09
bta-miR-22-3p	1.05	1.30E-22	bta-miR-EIA19-17220	3.78	8.68E-09
bta-miR-363	−1.60	2.13E-22	bta-miR-370	3.68	9.21E-06
bta-miR-EIA8-45133	−1.98	8.62E-20	bta-miR-142-5p	3.56	5.49E-11
bta-miR-2887	3.58	7.32E-18	bta-miR-2284w	3.55	1.14E-09
bta-miR-EIA6-40484	3.87	4.51E-17	bta-miR-142-3p	3.44	3.26E-07
bta-miR-EIA19-17190	3.29	5.78E-16	bta-miR-301a	3.39	1.53E-09
bta-miR-505	−1.64	9.27E-16	bta-miR-EIA4-36127	3.21	1.38E-06
bta-miR-193b	−1.93	1.94E-14	bta-miR-EIA13-8250	3.20	5.20E-06
bta-miR-574	−1.37	8.34E-14	bta-miR-EIA23-25367	3.17	7.04E-06
bta-miR-EIA6-40693	−2.00	1.90E-13	bta-miR-2484	3.17	1.19E-07
bta-miR-155	−1.55	4.75E-13	bta-miR-138	3.16	3.58E-06
bta-miR-7859	1.32	4.81E-13	bta-miR-2349	3.06	4.77E-05
bta-miR-744	−1.60	5.23E-13	bta-miR-6518	3.05	3.40E-05
bta-miR-EIA29-32291	3.20	1.05E-12	bta-miR-EIA7-42699	3.05	1.84E-08
bta-miR-EIA28-31634	2.16	1.44E-12	bta-miR-EIA1-612	3.03	3.45E-05
bta-miR-15b	−1.56	1.66E-12	bta-miR-EIA1-1319	3.03	3.45E-05
bta-miR-29d-3p	2.29	4.76E-12	bta-miR-EIA7-42410	3.02	4.94E-05
bta-miR-499	1.83	8.07E-12	**Involution and lactogenesis stages**		
bta-miR-582	−3.07	1.62E-10	bta-miR-2285t	−2.65	7.18E-24
bta-miR-EIA15-10725	3.06	1.46E-05	bta-miR-EIA8-45133	−2.68	4.53E-21
bta-miR-EIA29-31982	3.01	3.25E-05	bta-miR-EIA6-40693	−3.16	8.56E-19
bta-miR-EIAX-48475	−3.31	8.87E-05	bta-miR-885	3.78	1.70E-17
**Involution and galactopoiesis stages**			bta-miR-363	−1.74	4.73E-17
bta-miR-874	2.96	9.37E-14	bta-miR-490	−4.03	1.90E-16
bta-miR-149-5p	2.38	1.87E-13	bta-miR-29a	1.42	9.08E-15
bta-miR-222	3.71	1.87E-13	bta-miR-29b	2.49	3.76E-14
bta-miR-2387	2.09	3.52E-13	bta-miR-10b	3.74	6.60E-14
bta-miR-455-3p	2.92	1.49E-12	bta-miR-28	−1.32	8.72E-13
bta-miR-221	3.46	3.67E-12	bta-miR-EIA4-36830	−2.56	1.32E-12
bta-miR-361	1.09	4.04E-12	bta-miR-6524	−1.98	4.09E-12
bta-miR-330	2.96	8.71E-12	bta-miR-542-5p	3.39	1.41E-05
bta-miR-199a-5p	4.35	2.58E-10	bta-miR-EIA6-40484	3.17	2.84E-08
bta-miR-EIA1-391	4.34	4.10E-08	bta-miR-2887	3.02	1.47E-08

*^1^Top most significant miRNAs are those with a p-value BH ≤ 1.00E-12 or with a log2 fold change ≥3.00 and p-value BH ≤ 1.00E-5, ^2^Log2fold change.

**Table 4:** Dynamically differentially expressed miRNAs throughout the bovine lactation curve.

miRNAs/Stages^1^	GAL_vs_LAC		INV_vs_LAC		INV_vs_GAL		Expression	Pearson correlation with milk yield	
	L2FC^3^	p-value BH	L2FC	p-value BH	L2FC	p-value BH	Pattern^2^	R	p-value BH
bta-miR-106a	−0.73	3.55E-07	−1.1	1.14E-09	−0.36	1.93E-02	D_D_D	0.073	0.567
bta-miR-148b	−0.49	1.65E-02	−0.99	1.35E-04	−0.5	1.98E-02	D_D_D	−0.034	0.792
bta-miR-EIA15-10681	−0.44	4.73E-02	−0.95	9.00E-04	−0.51	3.82E-02	D_D_D	0.105	0.408
bta-miR-EIA16-12736	−1.77	3.94E-07	−2.65	1.65E-08	−0.88	4.23E-02	D_D_D	0.206	0.102
bta-miR-183	−0.69	2.42E-02	−2.33	2.87E-10	−1.64	1.40E-07	D_D_D	0.245	0.051
bta-miR-19b	−0.47	4.39E-03	−0.91	1.19E-05	−0.44	1.06E-02	D_D_D	0.106	0.406
bta-miR-EIA2-20521	−1.45	2.95E-06	−2.64	1.51E-10	−1.19	1.45E-03	D_D_D	0.102	0.424
bta-miR-224	−0.79	4.18E-03	−1.49	2.56E-05	−0.69	1.87E-02	D_D_D	0.004	0.975
bta-miR-2285t	−1.43	1.99E-11	−2.65	7.18E-24	−1.22	9.32E-08	D_D_D	0.272	0.029
bta-miR-EIA26-29519	−0.49	6.48E-03	−0.89	1.29E-04	−0.4	4.62E-02	D_D_D	0.072	0.573
bta-miR-28	−0.75	6.08E-07	−1.32	8.72E-13	−0.57	3.35E-04	D_D_D	0.036	0.780
bta-miR-EIA4-36830	−0.92	1.19E-03	−2.56	1.32E-12	−1.64	2.29E-07	D_D_D	0.305	0.014
bta-miR-490	−2.38	3.52E-11	−4.03	1.90E-16	−1.65	3.30E-04	D_D_D	0.144	0.256
bta-miR-EIA6-40693	−2	1.90E-13	−3.16	8.56E-19	−1.16	4.02E-04	D_D_D	0.108	0.396
bta-miR-EIA8-45133	−1.98	8.62E-20	−2.68	4.53E-21	−0.7	8.00E-03	D_D_D	0.339	0.006
bta-miR-6524	−0.52	3.02E-02	−1.98	4.09E-12	−1.46	1.84E-09	D_D_D	0.124	0.330
bta-miR-7862	−0.82	1.20E-02	−1.79	3.43E-05	−0.97	9.37E-03	D_D_D	0.163	0.199
bta-miR-146b	−2.15	7.61E-11	−1.33	2.44E-03	0.82	2.25E-02	D_U_D	−0.160	0.207
bta-miR-582	−3.07	1.62E-10	−1.49	2.27E-02	1.58	2.68E-03	D_U_D	−0.310	0.013
bta-miR-744	−1.6	5.23E-13	−0.8	7.54E-03	0.8	9.75E-04	D_U_D	−0.290	0.020
bta-miR-10a	−1.17	3.18E-02	1.6	2.70E-02	2.77	6.28E-07	D_U_U	−0.310	0.013
bta-miR-149-5p	−1.37	8.11E-06	1.01	1.45E-02	2.38	1.87E-13	D_U_U	−0.292	0.019
bta-miR-EIA19-19133	−1.79	1.28E-02	2.24	5.27E-03	4.03	2.54E-09	D_U_U	−0.399	0.001
bta-miR-221	−1.41	3.49E-03	2.05	8.97E-04	3.46	3.67E-12	D_U_U	−0.439	<0.001
bta-miR-222	−1.58	1.06E-03	2.13	6.45E-04	3.71	1.87E-13	D_U_U	−0.372	0.002
bta-miR-2323	−1.48	1.40E-02	1.47	4.84E-02	2.94	4.97E-07	D_U_U	−0.406	0.001
bta-miR-2331-3p	−1.47	1.37E-02	1.44	2.76E-02	2.91	1.34E-07	D_U_U	−0.326	0.008
bta-miR-27a-3p	−0.72	1.52E-02	1.28	6.45E-04	2	4.04E-11	D_U_U	−0.343	0.006
bta-miR-27a-5p	−0.75	3.83E-02	1.18	1.16E-02	1.93	1.19E-07	D_U_U	−0.410	0.001
bta-miR-330	−1.66	6.96E-05	1.3	1.88E-02	2.96	8.71E-12	D_U_U	−0.337	0.006
bta-miR-3432a	−0.77	4.39E-03	1.04	2.70E-03	1.81	6.95E-11	D_U_U	−0.446	<0.001
bta-miR-425-5p	−0.6	1.37E-03	0.57	2.51E-02	1.17	1.76E-09	D_U_U	−0.318	0.011
bta-miR-454	−1.12	7.65E-03	1.28	1.66E-02	2.4	9.96E-09	D_U_U	−0.461	0.000
bta-miR-455-3p	−1.62	4.31E-05	1.3	1.27E-02	2.92	1.49E-12	D_U_U	−0.202	0.109
bta-miR-EIA5-37953	−0.76	4.69E-02	1.03	3.22E-02	1.79	1.77E-06	D_U_U	−0.331	0.008
bta-miR-6529a	−0.68	2.05E-02	1.21	9.51E-04	1.89	1.53E-10	D_U_U	−0.466	<0.001
bta-miR-760-3p	−0.63	1.53E-02	1	2.02E-03	1.63	3.77E-10	D_U_U	−0.460	<0.001
bta-miR-877	−1.04	1.71E-04	0.86	1.87E-02	1.9	3.72E-11	D_U_U	−0.314	0.011
bta-miR-152	0.51	3.83E-02	−0.65	4.84E-02	−1.15	3.67E-06	U_D_D	0.334	0.007
bta-miR-EIA19-17190	3.29	5.78E-16	2.08	7.04E-05	−1.21	2.57E-03	U_D_U	0.149	0.241
bta-miR-193a-3p	1.63	7.92E-10	0.96	6.21E-03	−0.67	1.35E-02	U_D_U	0.135	0.288
bta-miR-22-5p	1.11	4.67E-10	0.73	1.84E-03	−0.38	4.72E-02	U_D_U	−0.038	0.764
bta-miR-2285o	1.8	5.77E-08	1.09	1.34E-02	−0.7	3.61E-02	U_D_U	0.168	0.184
bta-miR-29c	2.77	2.34E-26	2.1	4.13E-10	−0.68	1.89E-02	U_D_U	0.231	0.067
bta-miR-3064	2.63	1.26E-07	1.77	2.95E-03	−0.86	1.98E-02	U_D_U	0.121	0.340
bta-miR-30d	0.82	1.71E-07	0.43	4.64E-02	−0.38	2.25E-02	U_D_U	0.067	0.601
bta-miR-499	1.83	8.07E-12	1.11	1.62E-03	−0.72	9.65E-03	U_D_U	0.182	0.151
bta-miR-10b	2.71	3.06E-11	3.74	6.60E-14	1.03	1.44E-02	U_U_U	−0.332	0.007
bta-miR-133a	1.37	1.13E-02	2.28	1.72E-04	0.91	3.65E-02	U_U_U	−0.226	0.073
bta-miR-145	1.39	4.45E-04	2.65	6.01E-08	1.25	2.14E-03	U_U_U	−0.310	0.013
bta-miR-154c	1.22	7.29E-03	2.3	1.41E-05	1.08	7.90E-03	U_U_U	−0.181	0.151
bta-miR-EIA21-23041	2.31	2.70E-02	3.61	1.39E-03	1.3	4.81E-02	U_U_U	−0.175	0.166
bta-miR-23a	0.36	2.55E-02	1.15	4.90E-09	0.79	2.11E-06	U_U_U	−0.365	0.003
bta-miR-409a	1.38	1.41E-02	2.69	7.04E-05	1.31	1.35E-02	U_U_U	−0.180	0.154
bta-miR-421	0.35	6.16E-03	0.69	1.19E-05	0.35	8.24E-03	U_U_U	−0.257	0.040
bta-miR-493	1.34	1.29E-02	2.52	6.93E-05	1.19	1.38E-02	U_U_U	−0.232	0.065
bta-miR-6120-3p	0.68	5.67E-06	1.06	1.26E-08	0.38	1.63E-02	U_U_U	−0.373	0.002
bta-miR-9-3p	0.8	4.39E-02	1.93	3.51E-05	1.13	1.94E-03	U_U_U	−0.336	0.007

^1^GAL- galactopoiesis, LAC- lactogenesis, INV- involution.

^2^D: down regulated, U: Up regulated.

^3^Log2fold change.

**Table 5:** MiRNAs specifically significantly differentially expressed in each lactation stage*.

Lactation Stages^1^	miRNA	Log2 fold change	p-value BH
LAC	bta-miR-205	−2.28	1.01E-11
LAC	bta-miR-196a	−1.93	1.67E-08
LAC	bta-miR-92b	−0.77	5.75E-07
LAC	bta-miR-29d-5p	−0.68	2.09E-06
LAC	bta-miR-2285ad	0.43	8.29E-05
LAC	bta-miR-EIA19-16936	0.60	2.10E-04
LAC	bta-miR-346	−2.82	2.71E-04
LAC	bta-miR-380-3p	−1.71	3.29E-04
LAC	bta-miR-382	−1.66	7.46E-04
LAC	bta-miR-EIA28-30929	0.54	7.46E-04
LAC	bta-miR-EIA5-37717	0.55	7.46E-04
GAL	bta-miR-EIA23-25909	−1.56	3.82E-05
GAL	bta-miR-EIA13-8170	−1.91	9.09E-05
GAL	bta-miR-EIA26-29685	−1.46	2.17E-04
GAL	bta-miR-EIA7-43353	−0.80	2.57E-04
GAL	bta-miR-218	−1.39	5.44E-04
GAL	bta-miR-EIA17-14412	−0.81	6.25E-04
GAL	bta-miR-106b	−0.49	6.82E-04
GAL	bta-miR-EIA10-2785	−1.10	7.54E-04
GAL	bta-miR-EIAX-48106	−0.66	7.63E-04
GAL	bta-miR-2285i	−0.75	8.88E-04
GAL	bta-miR-339b	0.38	9.67E-04
INV	bta-miR-2284j	−1.42	3.85E-06
INV	bta-miR-182	−1.16	7.16E-06
INV	bta-miR-EIA20-22101	−1.25	2.64E-05
INV	bta-miR-452	−1.30	2.35E-04
INV	bta-let-7a-5p	−0.54	4.29E-04
INV	bta-miR-EIA1-1056	−1.05	4.87E-04
INV	bta-miR-EIA20-21802	1.09	5.33E-04
INV	bta-miR-30f	−1.03	5.45E-04
INV	bta-miR-2419-5p	0.79	7.80E-04

^*^Only miRNAs with p-value BH < 0.001 are shown, ^1^GAL- galactopoiesis, LAC- lactogenesis, INV- involution.
