# Supplementary material for: MicroRNA roles in signalling during lactation: an insight from differential expression, time course and pathway analyses of deep sequence data
Source: Sci Rep. 2017 Mar 20;7:44605. doi: 10.1038/srep44605 (PMC5357959; doi:10.1038/srep44605)
Supplement: Supplementary Information [file srep44605-s1.pdf]

MicroRNA roles in signaling during lactation: an insight from differential expression, time course and pathway analyses of deep sequence data

Duy N. Do[1, 2], Ran Li[1, 3], Pier-Luc Dudemaine[1] and Eveline M. Ibeagha-Awemu[1*]

[1]Agriculture and Agri-Food Canada, Sherbrooke Research and Development Centre, 2000 College Street, Sherbrooke, Quebec, J1M 0C8, Canada

[2]Department of Animal Science, McGill University, 21111, Lakeshore Road, Ste-Anne-de Bellevue, Quebec, J1M 0C8, Canada

[3]College of Animal Science and Technology, Northwest A&F University, Xinong road 22, Shaanxi, 712100, China

[*]**Corresponding author:**

Dr. Eveline Ibeagha-Awemu

Agriculture and Agri-Food Canada

Sherbrooke Research and Development Center, Sherbrooke, QC, Canada

Eveline.ibeagha-awemu@agr.gc.ca

Supplementary figures

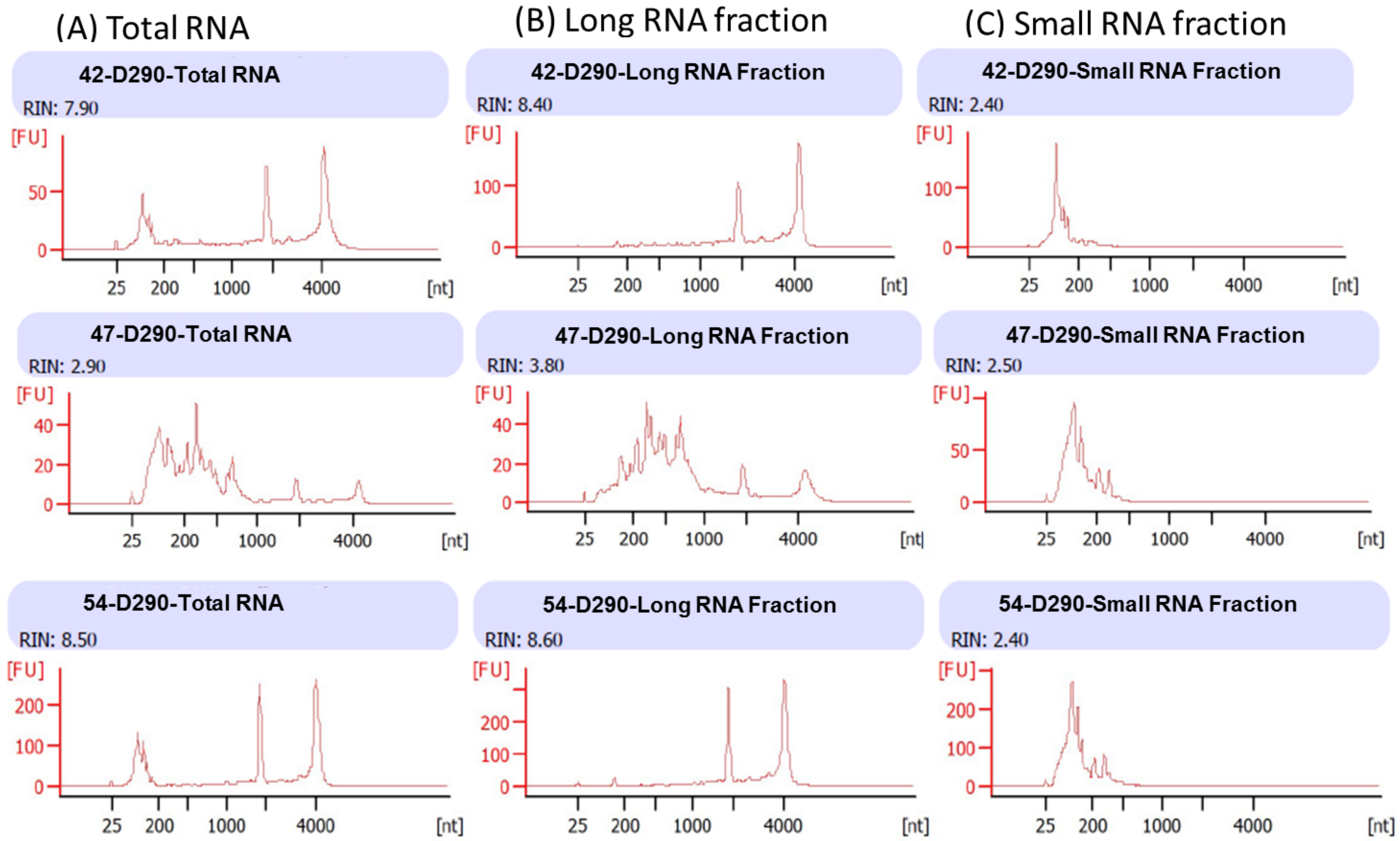

Figure S1. The quality of RNA isolated from milk fat samples. The RIN (RNA integrity number) values ranged from 2.3 to 8.5. To see if the small RNA fraction was intact, we enriched the small RNA fraction using MiRVana kit which allows for the simultaneous extraction/enrichment of long RNA (>200bps) and small RNA (<200bp) fractions from the same sample. Analysis of RIN following enrichment showed an improvement in the RIN values of the long RNA fraction (B), while a sharp peak between nucleotide 25 and 200 showed that the small RNA fraction was intact (C). Furthermore, the RIN value of enriched RNA fractions (small and large) (B, C), depends on the quality of the starting material (A). As shown in figure, the RIN value of the starting total RNA was 7.9 (sample 42) while that of the long RNA fraction was 8.4 (E) and of the small RNA fraction, 2.4 (F). Since the small RNA fraction was intact, RNA used in library preparation in this study was not enriched. It has been shown that, some small RNA species are lost following the enrichment step (Podolska *et al*. 2011. How the RNA isolation method can affect microRNA microarray results. Acta Biochimica Polinica 58: 535-540, http://www.actabp.pl/pdf/4_2011/535.pdf)

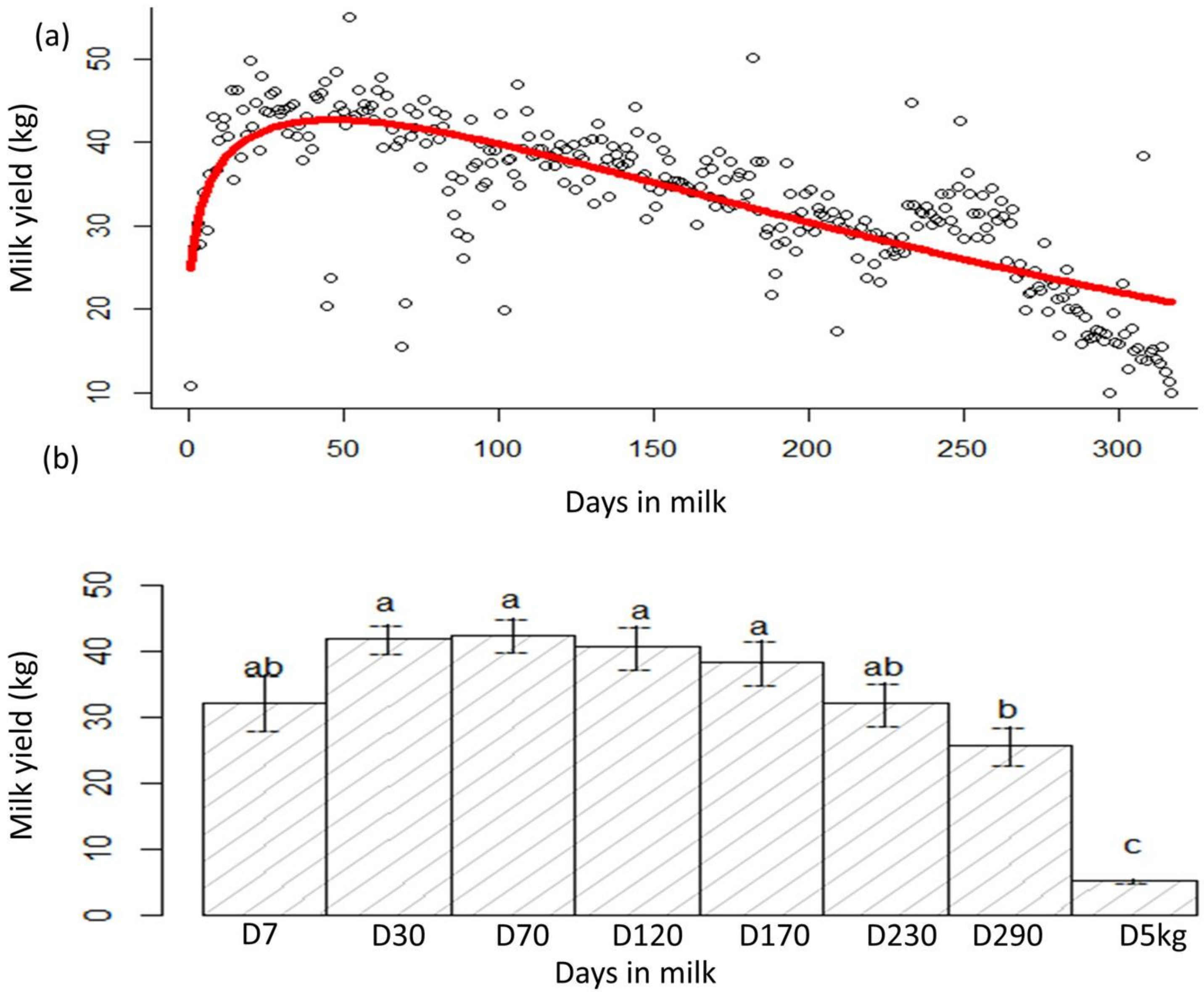

Figure S2. Daily milk yield and differences in milk yield between lactation days. (a) Daily raw milk yield (circles) and predicted lactation curve for milk yield using Wood's model (red curve). (b) Tukey's HSD (honest significant difference) test for effects of day on milk yield. [a,b,c]Means with different letters differ significantly at $p<0.05$.

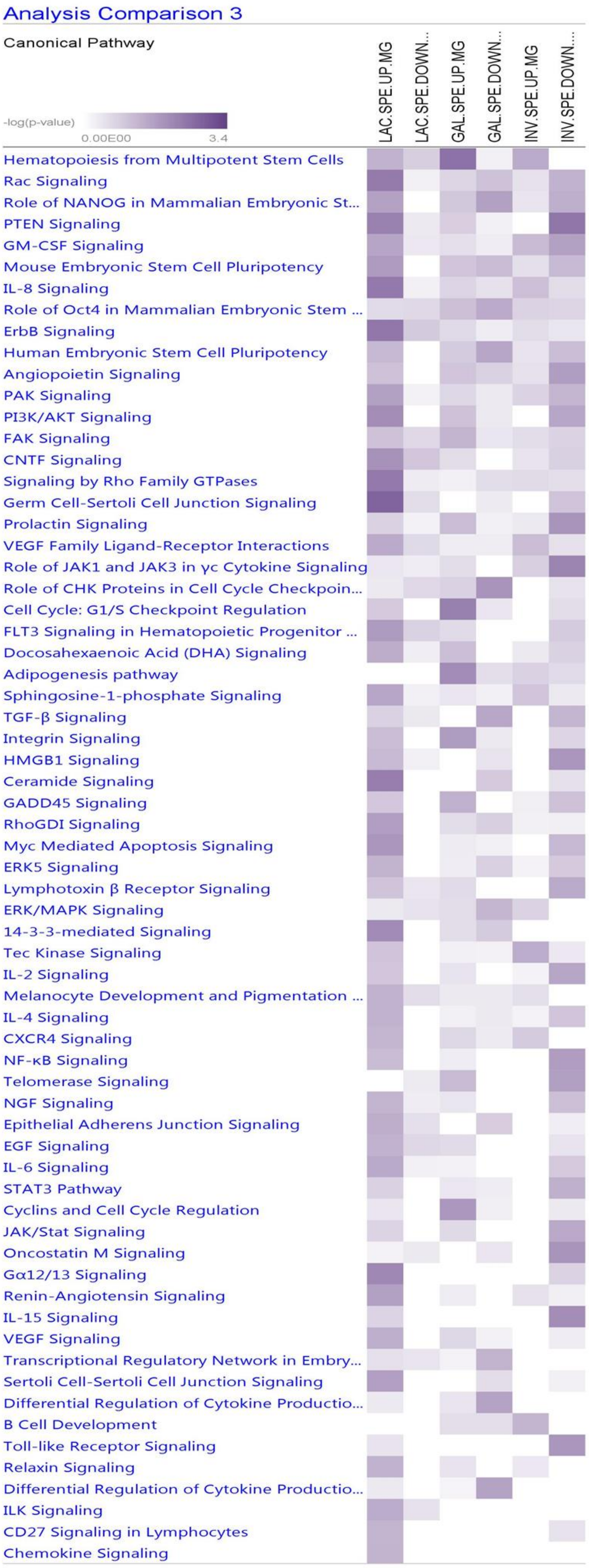

Figure S3. Signalling pathways enriched for target genes of differentially expressed miRNAs specific to each lactation transition stage. LAC: Lactogenesis; GAL: Galactopoiesis and INV: Involution

Supplementary Table Titles

Table S1. Mapping statistics of miRNA-Seq reads

Table S2. Length (nt) distribution of miRNA reads

Table S3. Know and novel miRNA read counts

TableS4. Summary of MiRDeep2 output for miRNA prediction

Table S5. Highly expressed miRNAs during different stages of the bovine lactation curve and constituted 78.2, 84.2 and 81.4% of all read counts in LAC, GAL and INV stages, respectively. LAC: Lactogenesis; GAL: Galactopoiesis and INV: Involution

TableS6. Pathways enriched for target genes of 15 abundantly expressed miRNAs across lactation stages

Table S7. Molecular and cellular functions (a), physiological system development functions (b) and pathways (c) enriched for target genes of miR-EIA3-33361 (a novel miRNA)

Table S8. Significantly differentially expressed miRNAs between (a) galactopoiesis and lactogenesis, (b) involution and galactopoiesis, (c) involution and lactogenesis stages and (d) selected differentially expressed miRNAs for downstream analyses

Table S9. Pathways enriched for target genes of the most significantly differentially expressed miRNAs at each lactation stage: a) miR-29b, b) miR-363, c) miR-874, d) miR-6524, e) miR-885 and f) miR-2285t

Table S10. Enriched (a) molecular and cellular functions and (b) physiology system development functions for target genes of differentially expressed miRNAs for each lactation stage comparison

Table S11. Enriched canonical pathways for target genes of differentially expressed miRNAs for each lactation stage comparison

Table S12. Enriched pathways for dynamic differentially expressed miRNAs throughout the bovine lactation curve

Table S13. Stage specific differently expressed miRNAs.

Table S13. Stage specific differently expressed miRNAs

Stage	miRNA	baseMean	log2FoldChange	pvalue	padj
GAL	**bta-miR-23-25909**	1.73	-1.56	9.24E-06	3.82E-05
GAL	bta-miR-13-8170	2.39	-1.91	2.45E-05	9.09E-05
GAL	bta-miR-26-29685	1.74	-1.46	6.6E-05	0.000217
GAL	bta-miR-7-43353	8.86	-0.80	8.16E-05	0.000257
GAL	bta-miR-218	2.68	-1.39	0.000193	0.000544
GAL	bta-miR-17-14412	9.57	-0.81	0.000228	0.000625
GAL	bta-miR-106b	137.15	-0.49	0.000251	0.000682
GAL	bta-miR-10-2785	2.98	-1.10	0.000279	0.000754
GAL	bta-miR-X-48106	28.82	-0.66	0.000284	0.000763
GAL	bta-miR-2285i	14.12	-0.75	0.000337	0.000888
GAL	**bta-miR-339b**	2241.99	0.38	0.000372	9.67E-04
GAL	bta-miR-6-40377	2.55	-1.03	0.000419	0.001072

GAL	bta-miR-9-46001	2.55	-1.03	0.000419	0.001072
GAL	bta-miR-8-43973	5.43	0.79	0.000679	0.001635
GAL	bta-miR-2285v	12.06	-0.64	0.000797	0.001883
GAL	bta-miR-21-3p	13.13	-1.27	0.000981	0.002243
GAL	bta-miR-12-6501	34.11	0.46	0.001228	0.002767
GAL	bta-miR-190a	11.71	0.90	0.001374	0.00305
GAL	bta-miR-2284z	28.70	-0.56	0.001545	0.003336
GAL	bta-miR-374a	4774.64	0.45	0.001645	0.003528
GAL	bta-miR-345-5p	249.05	0.43	0.001684	0.003599
GAL	bta-miR-2284o	4.82	0.80	0.001906	0.004032
GAL	bta-miR-3-34194	34.50	0.42	0.002429	0.005035
GAL	bta-miR-24-27504	1.09	-1.32	0.002477	0.005068
GAL	bta-miR-12-6619	1.32	-1.59	0.002841	0.005794
GAL	bta-miR-2447	4.84	-0.75	0.003135	0.006332
GAL	bta-miR-X-48418	9.72	-0.56	0.003589	0.007133
GAL	bta-miR-2398	0.62	-1.89	0.004378	0.008538
GAL	bta-miR-2454-3p	1.28	-1.39	0.004661	0.009004
GAL	bta-miR-16-12836	17.43	-0.41	0.005402	0.010339
GAL	bta-miR-X-48631	73.89	0.46	0.006588	0.012457
GAL	bta-miR-655	1.09	1.31	0.006789	0.01276
GAL	bta-miR-2285e	40.69	0.35	0.007601	0.014115
GAL	bta-miR-4-36279	4.23	-0.71	0.007988	0.01479
GAL	bta-miR-22-25039	9.74	0.48	0.008717	0.016045
GAL	bta-miR-10-2226	4.14	-0.67	0.009136	0.016768
GAL	bta-miR-19-18310	1.96	1.06	0.009281	0.016964
GAL	bta-miR-1301	0.91	-1.25	0.011338	0.019929
GAL	bta-miR-708	4.68	-0.86	0.011716	0.020536
GAL	bta-miR-6517	28.63	-0.40	0.01291	0.022377
GAL	bta-miR-7-42243	0.68	-2.09	0.01383	0.023708
GAL	bta-miR-24-27529	5.80	0.58	0.014194	0.024266
GAL	bta-miR-25-28019	1.23	-1.09	0.014378	0.024514
GAL	bta-miR-2336	26.71	-0.41	0.014908	0.025348
GAL	bta-miR-122	1.61	1.04	0.015116	0.025631
GAL	bta-miR-2-20813	2.43	0.93	0.016203	0.027327
GAL	bta-miR-20a	2615.11	0.25	0.016486	0.027669
GAL	bta-miR-13-8608	1.03	1.21	0.016569	0.027719
GAL	bta-miR-26-29785	1.28	-1.36	0.01712	0.028564
GAL	bta-miR-24-27349	3.55	0.54	0.022565	0.03686
GAL	bta-miR-320a	6714.72	0.50	0.026888	0.042693
GAL	bta-miR-17-13951	174.60	0.53	0.027686	0.043626
GAL	bta-miR-30e-5p	18658.43	0.20	0.030386	0.047641
INV	**bta-miR-2284j**	15.59	-1.42	2.47E-07	3.85E-06
INV	bta-miR-182	6974.23	-1.16	5.5E-07	7.16E-06
INV	bta-miR-20-22101	624.88	-1.25	2.67E-06	2.64E-05
INV	bta-miR-452	13.17	-1.30	3.36E-05	0.000235
INV	bta-let-7a-5p	152078.85	-0.54	6.4E-05	0.000429
INV	bta-miR-1-1056	14.33	-1.05	7.56E-05	0.000487
INV	**bta-miR-20-21802**	4.34	1.09	8.79E-05	5.33E-04
INV	bta-miR-30f	12903.58	-1.03	9.35E-05	0.000545
INV	bta-miR-2419-5p	160.74	0.79	0.000145	0.00078
INV	bta-miR-2483-3p	1.38	1.35	0.000595	0.002526
INV	bta-miR-23b-3p	4938.57	-0.41	0.002268	0.007691
INV	bta-miR-28-31754	13.59	-0.97	0.002427	0.008097
INV	bta-miR-2903	2.34	-1.39	0.002855	0.00917
INV	bta-miR-1306	16.52	1.15	0.002886	0.00917
INV	bta-miR-3-34195	5.81	-0.73	0.003046	0.00955
INV	bta-miR-331-3p	277.56	0.46	0.003117	0.009725
INV	bta-miR-5-39289	0.88	-3.13	0.004109	0.012328
INV	bta-miR-30b-3p	34.21	-0.52	0.004275	0.012703

INV	bta-miR-665	1.01	1.40	0.005519	0.015374
INV	bta-miR-2443	10.96	-0.89	0.005624	0.015547
INV	bta-let-7g	24480.19	-0.38	0.007233	0.018807
INV	bta-miR-3-32952	17.70	-0.69	0.008754	0.022572
INV	bta-miR-5-39610	6.50	-1.21	0.011302	0.028098
INV	bta-miR-3-34571	3.58	0.78	0.011776	0.029045
INV	bta-miR-2435	2.05	-1.09	0.017555	0.03998
INV	bta-miR-12-6940	174.04	-0.79	0.017511	0.03998
INV	bta-miR-11-4652	1.32	1.13	0.017756	0.040289
INV	bta-miR-X-48416	163.32	-0.78	0.018142	0.041017
INV	bta-miR-484	724.87	0.59	0.018459	0.041432
INV	bta-miR-345-3p	183.46	-0.33	0.020356	0.044568
INV	bta-miR-6-40066	8.14	0.57	0.021159	0.046005
LAC	**bta-miR-205**	79.20	-2.28	3.25E-13	1.01E-11
LAC	bta-miR-196a	161.17	-1.93	8.85E-10	1.67E-08
LAC	bta-miR-92b	244.01	-0.77	4.33E-08	5.75E-07
LAC	bta-miR-29d-5p	95.86	-0.68	1.71E-07	2.09E-06
LAC	**bta-miR-2285ad**	37.87	0.43	1.04E-05	8.29E-05
LAC	bta-miR-19-16936	23.13	0.60	2.76E-05	0.00021
LAC	bta-miR-346	1.64	-2.82	3.78E-05	0.000271
LAC	bta-miR-380-3p	6.43	-1.71	4.64E-05	0.000329
LAC	bta-miR-382	4.93	-1.66	0.000125	0.000746
LAC	bta-miR-28-30929	24.73	0.54	0.000125	0.000746
LAC	bta-miR-5-37717	25.77	0.55	0.000125	0.000746
LAC	bta-miR-21-23724	6.31	1.03	0.000367	0.001753
LAC	bta-miR-652	2013.00	-0.45	0.000454	0.002037
LAC	bta-miR-2299-3p	5.46	1.04	0.000557	0.00238
LAC	bta-miR-128	468.52	0.28	0.001951	0.007227
LAC	bta-miR-451	6.39	2.59	0.003678	0.012142
LAC	bta-miR-2299-5p	10.14	0.93	0.004317	0.013814
LAC	bta-miR-1	10.09	-1.27	0.004574	0.014342
LAC	bta-miR-2-19312	13.13	0.87	0.004647	0.014499
LAC	bta-miR-432	4.36	-1.24	0.004781	0.014842
LAC	bta-miR-181a	8315.90	0.50	0.004921	0.015201
LAC	bta-miR-8-43999	1.39	-1.60	0.006078	0.018501
LAC	bta-miR-13-8780	1.53	1.44	0.007912	0.022753
LAC	bta-let-7f	36225.40	0.43	0.010248	0.028548
LAC	bta-miR-12-6781	0.76	1.26	0.015405	0.040053
LAC	bta-miR-2436-3p	2.91	-1.02	0.016825	0.043027
LAC	bta-miR-103	13113.09	-0.26	0.019341	0.048469
LAC	bta-miR-2284a	1.18	1.04	0.019813	0.049453

LAC: Lactogenesis

GAL: Galactopoiesis

INV: Involution

Table S14. Enriched pathways for time dependent differentially expressed miRNAs throughout the bovine lactation curve
